# Chemical space exploration based on recurrent neural networks: applications in discovering kinase inhibitors

**DOI:** 10.1186/s13321-020-00446-3

**Published:** 2020-06-08

**Authors:** Xuanyi Li, Yinqiu Xu, Hequan Yao, Kejiang Lin

**Affiliations:** Department of Medicinal Chemistry, School of Pharmacy, China Pharmaceutical University, 24 Tongjiaxiang, Nanjing, 210009 China

**Keywords:** Recurrent neural networks, De novo molecular generation, Chemical space, Kinase inhibitors

## Abstract

With the rise of artificial intelligence (AI) in drug discovery, de novo molecular generation provides new ways to explore chemical space. However, because de novo molecular generation methods rely on abundant known molecules, generated molecules may have a problem of novelty. Novelty is important in highly competitive areas of medicinal chemistry, such as the discovery of kinase inhibitors. In this study, de novo molecular generation based on recurrent neural networks was applied to discover a new chemical space of kinase inhibitors. During the application, the practicality was evaluated, and new inspiration was found. With the successful discovery of one potent Pim1 inhibitor and two lead compounds that inhibit CDK4, AI-based molecular generation shows potentials in drug discovery and development.
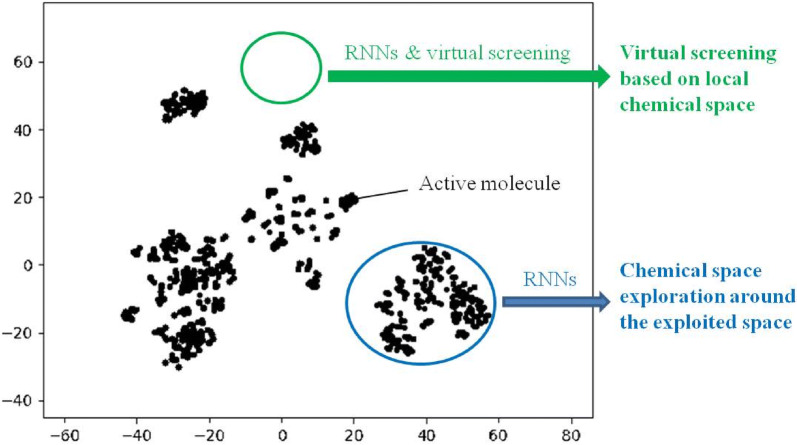

## Introduction

Chemical space is defined as the infinite universe of molecules [1], where unknown space is being explored and developed. Considering only drug-like molecules, the number of compounds in the drug-like chemical space is estimated to be 10^60^, which means that there are more drug-like compounds than there are atoms in the solar system [2]. In the drug-like chemical space, only a tiny proportion of molecules have been found as drugs, and for a long time, numerous efforts have been made to modify the drug map. After years of high-cost and time-consuming trials exploring the drug space, computers started to be used to guide the exploration in the 1980s, when computer-assisted drug design (CADD) emerged [3]. However, even with the help of quantum approaches, which require massive calculation, the biological activity of compounds can hardly be predicted precisely. As no formula can be found to precisely describe the interactions between molecules and their targets, automating drug research and development (R&D) through machine learning (ML) from a large number of samples represents a new option [4, 5]. In recent years, explorers of the drug map have started to think differently, and they are turning to artificial intelligence (AI) as an effective guide [6–8].

Inspired by the successful applications of deep learning in areas such as image recognition and natural language processing [9], researchers have increased their interest in the deployment of AI in drug R&D [10]. As reviewed in several articles [10–14], deep learning (DL) and AI have had significant effects on CADD. Especially, the linear form of molecules is similar to sequences in natural language processing and thus establishes a starting point for de novo molecular generation.

De novo molecular generation aims to produce new chemical space with certain properties, which has been greatly bolstered by NN-based algorithms, as introduced recently [15]. Simplified molecular input line entry specification (SMILES) [16] and international chemical identifier (InChI) [17] are two linear representations of molecules that have been applied to de novo molecular generation, and SMILES is more commonly used due to its simple grammar. During training, RNN-based models try to learn how sequences of training molecules are composed, so the models are able to regenerate the sequences after training. And even, the models can further reorganize sequences, so to produce molecules that are structurally novel but similar to those known molecules. Although there are novel molecules generated, their fragments are mainly learned from the training molecules. To some degree, those structurally novel but similar molecules further enrich the chemical space around the training molecules, and the generated molecules represent the local chemical space around the training molecules. In early studies [18, 19], transfer learning (TL) was adopted by RNN-based models to generate the chemical space around target molecules quickly and effectively. During TL, models are first trained with datasets providing a large number of molecules. Then, the models will be fine-tuned with target molecules. TL performed on molecules resembles a process during which the learned chemical space is transferring from those unrelated datasets to desired molecules. With this method, researchers have successfully discovered several compounds with moderate to high activity [20]. Details of generative models based on NNs have been recently reviewed [15].

Although various complex models designed for de novo molecular generation have been created, their real performance in the exploitation of chemical space remains uncertain. Sometimes, complex models may not perform as well as expected [21]. In comparative studies [22, 23] on different generative models, simpler models such as models based on long short-term memory (LSTM) [24] are found to be more powerful than complex models, and simple RNNs with SMILES as inputs are one of those satisfactory models. Generative models based only on simple RNNs remember or generalize the chemical space around target molecules directly, so simple models have been found to be effective tools for exploring the chemical space around target molecules [25, 26]. Notably, generative models are usually trained with a large number of data, which in turn could be at the expense of losing novelty. With a background of strict protection of intellectual property (IP), such as Markush structures covered by IP, novelty and accuracy seem to be necessary issues for generative models [27]. To help relieve the uncertainty in the models, their application in real tasks seems indispensable.

Because the R&D of kinase inhibitors is part of a competitive field in medicinal chemistry, a successful trial of RNN-based models could be persuasive. Proviral integration site for Moloney murine leukemia virus kinase 1 (Pim1) and cyclin-dependent kinase 4 (CDK4) are two widely-studied kinases, and the online database ChEMBL [28] collects hundreds of known inhibitors for each of the two targets. In 2017, Abemaciclib was approved by the United States Food and Drug Administration (FDA), whose half maximal inhibitory concentration (IC_50_) values for Pim1 and CDK4 are 50 nM and 2 nM [29]. Additionally, both Pim1 inhibitors and CDK4 inhibitors show anti-tumor activities through affecting cell cycle [30, 31], and the two targets show potentials in treatment of renal cell carcinoma [32]. The both targets have abundant and potent inhibitors, namely, the chemical space of their inhibitors has been greatly exploited. Overall, exploring new chemical space of Pim1 inhibitors and CDK4 inhibitors is challenging, which helps test the performance of those generative models.

**Fig. 1:**
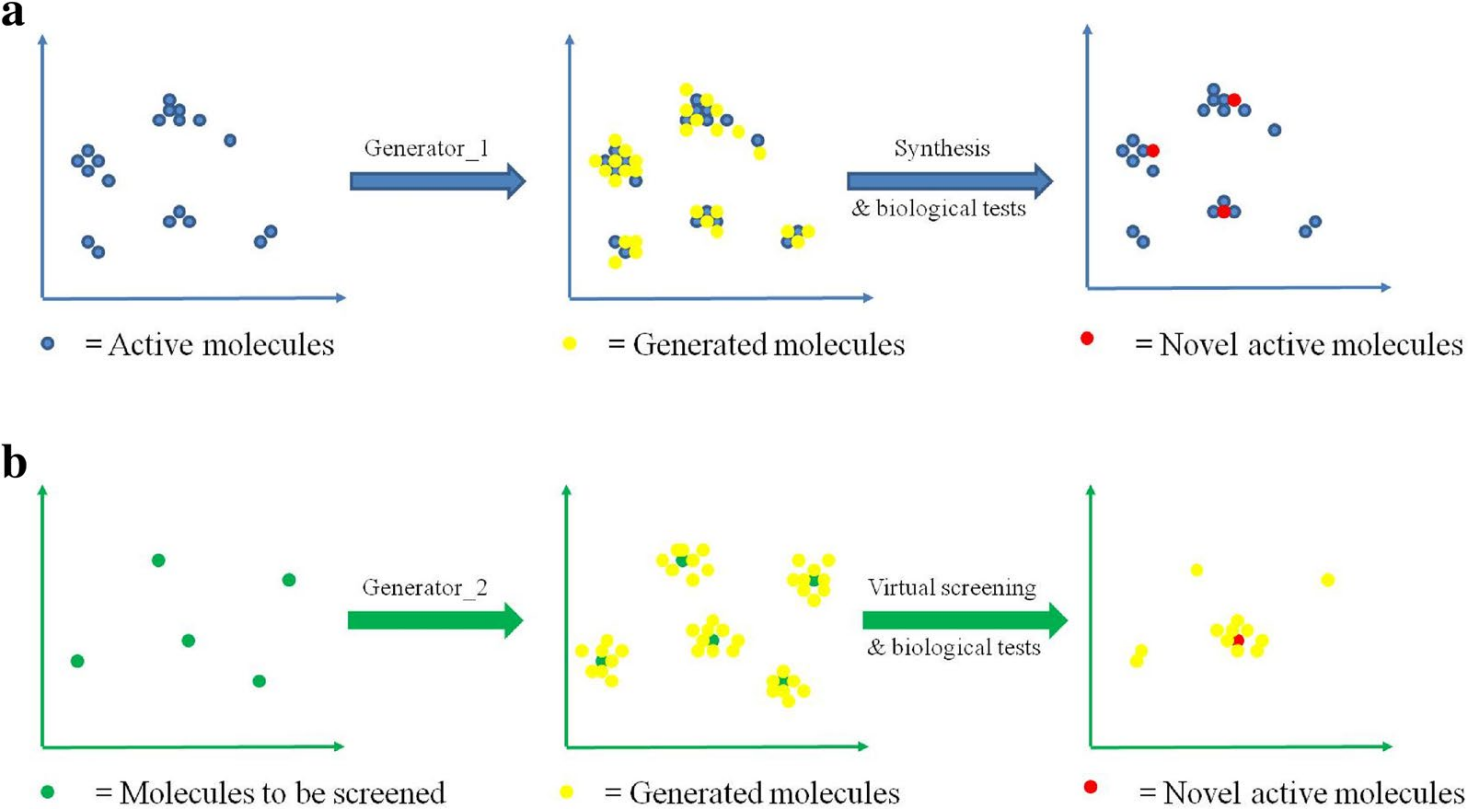
Chemical space exploration with RNNs. **a** Direct chemical space exploitation around known active molecules. Generator_1 is used to generate SMILES sequences, after being trained with SMILES sequences of active molecules. **b** Chemical space exploration for an unknown space. RNNs and virtual screening are combined to realize virtual screening based on local chemical space. Molecules are generated with generator_2, which has been trained through TL

In this study, we applied RNN-based generative models to generate potential inhibitors for Pim1 and CDK4. With RNN-based generative models, this study aims to explore spaces both near and far from the explored space. As shown in Fig. 1a, RNNs can be directly applied to generate molecules based on training molecules to realize exploration near the exploited space around the training molecules. However, the neighbor exploited space implies insufficient novelty. To solve this problem, we combined RNN-based generative models and virtual screening. According to structure–activity relationship (SAR) studies, the chemical space of active molecules could be local, so the larger local space enriched by virtual screening may correspond to a higher probability of discovering novel active molecules. As shown in Fig. 1b, RNNs were applied to molecular generation based on molecules to be screened, so virtual screening can be performed on the groups of similar molecules to the training molecules rather than the training molecules alone. The proposed idea was then validated in silico and in practice. During the application, details on the implementation are uncovered to help better improve the models used for de novo molecular generation. Finally, the models that we prepared successfully designed one potent Pim1 inhibitor and two novel lead compounds targeting CDK4.

## Methods

### Datasets prepared for generative models

All the molecules in our homemade datasets were downloaded from ChEMBL 24.1 (https://www.ebi.ac.uk/chembl/) [28]. Only inhibitors collected in the online database with certain IC_50_ values were considered, and the molecules were then sorted by their IC_50_ values from small to large. The first 500 molecules with smallest IC_50_ values were downloaded respectively as active molecules for the two targets. Without considering chirality, replicate molecules were then removed, and 448 CDK4 inhibitors and 453 Pim1 inhibitors were used for generator_1, generator_canonical and generator_random. TL was also applied in this study to pre-train generator_2, and the DrugBank 5.1.2 database [33] was downloaded. Molecules in DrugBank were sanitized, followed by removing their chirality with RDKit [34], and 7577 molecules were ultimately prepared.

SMILES sequences of CDK4 inhibitors, Pim1 inhibitors and drugs were then prepared with RDKit. All the molecules were sanitized, and their chirality was removed. Canonical SMILES sequences of the collected inhibitors were used to train the generator_canonical. At the same time, randomized SMILES sequences belonging to those inhibitors were used to train the generator_1 and the generator_random, and the sequences were prepared as described before [35]. Meanwhile, the randomized SMILES sequences belonging to preprocessed molecules collected in drugbank were used to train the generator_2. During the preparation for the randomized sequences, the atom ordering of every molecule was changed randomly to produce different SMILES sequences.

The SMILES sequences prepared for each model were mixed at random respectively before being input. Each sequence was ended with a “\n” symbol, and the sequences in each dataset were concatenated respectively without padding to a fixed length. The “\n” symbol separates two neighbor sequences, indicating both the end of a previous sequence and the start of the next sequence. The concatenated sequences were then divided into tokens. Tokens represent certain atoms, bonds and connections that appeared in SMILES sequences. In this study, the tokens used are c, C, n, N, o, O, s, S, p, P, F, I, 1, 2, 3, 4, 5, 6, 7, 8, −, +, [, ], (, ), =, #, \n, [nH], [S+], [O−], [N+], [N−], Br, Cl and Si.

In each dataset, tokens of all the sequences were divided into 128 batches, and 128 batches of continuous tokens were input through N times. Every time 64 tokens in each batch were input, and there were a total of 128 × 64 tokens being input. Namely, 128 batches of tokens were input for 64 time steps every time. Tokens that are not included in the N × 128 × 64 tokens through N times of inputs will be ignored. Before being input, the tokens were encoded with one-hot encoding. Next tokens of current tokens being input were the targets for prediction during training, which were represented with one-hot encoding as well.

### RNN-based generative models

All the models were built with TensorFlow 1.5.0 [36] as described before [19, 37]. The computations were performed in a Linux (Ubuntu 18.04) personal computer with CPU only. Because previous studies [18, 19] had reported appropriate values of loss function for molecular generation, which indicate satisfying molecular generation, in this study the loss values reported before were set a goal. Before final training of each model, hyperparameters were adjusted until the loss function approached values reported before.

In all the models, two stacked LSTM layers were used with the BasicLSTMCell function provided by Tensorflow, and dropout was used for outputs of each LSTM layer, with a keep probability of 0.8. The multi-LSTM layers were implemented by the dynamic_rnn function provided by Tensorflow. During each time step among the same epoch, the output states of the current time step were kept as the initial states for the next time step. The output of the multi-LSTM layers was finally connected to a fully connected layer, followed by conversion to probabilities with softmax. During training, cross entropy of sequences was used as the loss function. The loss function was implemented by the seq2seq.sequence_loss function, which was provided by Tensorflow to calculate loss for sequences. The loss function was optimized by theTensorFlow ADAM optimizer [38], with a learning rate of 0.003. Meanwhile, a gradient norm clipping of 5 was applied during training.

In the comparative study on the effects of diverse randomized SMILES sequences, generator_canonical and generator_random were trained, and there were 256 units in each LSTM layer. Generator_canonical was trained with canonical SMILES sequences of the inhibitors mentined in part 2.1, while generator_random was trained with those randomized SMILES sequences. Genarator_1 was trained directly with the randomized sequences of CDK4 inhibitors and Pim1 inhibitors to generate the chemical space around known inhibitors, and there were also 256 units in each layer. To perform virtual screening on the local chemical space around molecules to be screened, 512 units of each LSTM layer were used in generator_2. The generator_2 was pre-trained with the database preprocessed from DrugBank for 50 epochs, to improve the validity during molecule generation. The pretraining process took about 3 days, while the generator_1, the generator_canonical and the generator_random were trained within 8 h, respectively.

During the sampling of tokens, the “\n” symbol was used as the first token, and tokens of next steps were sampled continuously with previously predicted tokens and final states as new inputs and initial states. A total of 200,000 tokens were sampled randomly according to their predicted probability corresponding to each predefined token. Then, incorrect sequences and replicated molecules were removed with RDKit.

### Evaluations on the generated chemical space

The generated chemical space was first described with the similarity between the generated molecules and the training molecules. The Tanimoto similarity index was used, and circular fingerprints with a radius of 3 were used to represent the molecules. The calculations were implemented with RDKit.

T-distributed stochastic neighbor embedding (t-SNE) [39] is a powerful algorithm that helps visualize high-dimensional data to understand data structures. Molecules were represented with their circular fingerprints, which were then hashed into 1024-bit vectors. The 1024-bit vectors of the generated molecules were projected to 2-D space with t-SNE, which was performed with Scikit-Learn [40].

### Synthesis

Three molecules selected from the generated molecules produced by generator_1 were modified and synthesized. The synthesis of MJ-1055 was based on methods reported before [41]. Detailed operations and spectra can be found in Additional file 1.

### Pharmacophore models and molecular docking

Pharmacophore models and molecular docking were prepared to perform virtual screening based on the local chemical space

Active molecules and inactive molecules were prepared to validate the models. Active molecules were the 1000 molecules downloaded as described in part 2.1, while inactive molecules of CDK4 and Pim1 were downloaded from ChEMBL 24.1 with a “>” symbol indicating their lack of activity. The chirality of both active and inactive molecules was considered, and duplicates were removed, which resulted in 499 CDK4 inhibitors, 499 Pim1 inhibitors, 97 molecules inactive toward Pim1 and 53 molecules inactive toward CDK4. Both active molecules and inactive molecules used for validating pharmacophore models and molecular docking were prepared with the preparation ligand module in Discovery Studio 3.0 (DS) [42].

During the construction of those models, the specificity, sensitivity and area under the curve (AUC) were used to evaluate the models. The specificity and sensitivity of the models were calculated as follows:

$${\text{Specificity}} = {\text{true negative}}/\left( {{\text{true negative}} + {\text{false positive}}} \right)$$

$${\text{Sensitivity}} = {\text{true positive}}/\left( {{\text{true positive}} + {\text{false negative}}} \right)$$

The active molecules that are correctly predicted are defined as true positive, the inactive molecules that are correctly predicted are represented by true negative, and false positive and false negative are defined as their incorrectly predicted counterparts, respectively.

Because CDK4 and CDK6 are highly homologous and there is no crystal structure of CDK4 and its inhibitors, pharmacophore models of CDK4 inhibitors were built based on the complex structures of CDK6 and its inhibitors. The models were built with the receptor-ligand pharmacophore generation module in DS 3.0, and the receptor-ligand pharmacophore generation module has been introduced before [43]. During the building of models, the parameters were set as default. The Protein Data Bank (PDB) code of the complex structures used for modeling includes 5L2I, 5L2S, 5L2T, 4TTH, 4EZ5, 4AUA, 3NUX, 3NUP, 2F2C, and 2EUF.

A docking model was also built based on a crystal structure of CDK4 (PDB code: 2W96) to help improve the performance of screening. LigandFit [44] provided by DS was chosen as the docking method, and the cavity centered with a coordinate (x = 8.03, y = 3.06, z = 75.12) was defined as the binding site. After molecular docking, the specificity and sensitivity were first calculated, and then, scoring functions including DOCK_SCORE, LigScore1, LigScore2, -PLP1, -PLP2, Jain, LUDI, -PMF and -PMF04 were compared. The AUC values were calculated with the highest score among the poses of each molecule.

Pharmacophore models used to screen Pim1 inhibitors were built based on complex structures of Pim1 and its inhibitors, and the PDB codes include 4MBI, 1YXU, 4A7C, 4BZO, 4MBI, 4RPV, 4XHK, 5VUB, 2BIL, 2O63, 3T9I, 3UIX, 4ASO, 4BZN, 4I41, 4IAA, 4JX7, 4MED, 4RBL, 4RC2, 5NDT, 6BSK, 3DCV, 3F2A, 3JPV, 3MA3, 4LM5, 5KCX, 5OL1, 5TEL, 5TEX, 5TOE, 5TUR, 2XJZ, 3JXW, 3UMX, 5DIA, 5OY4, 5V80, 3VBQ, 4DTK, 4TYL, 1YSK, 4K18, 4WRS, 4WSY, 4WT6, 5DGZ, 5DWR, 5EOL, 5IIS, 5IPJ, 5KZI, 3BGP, 3BGQ and 4ENX. The number of minimum features was set to 3, and other parameters were set to default values.

### Virtual screening based on local chemical space

Traditional models treat molecules individually, which makes them sensitive to small structural modifications of molecules. Compared to one molecule being the screening result of the traditional models, a number of screened molecules with the same core structure but diverse structural modifications indicate the core structure suits the models better.

RNN-based generators are good at generating a group of molecules that are similar to a training molecule. To some degree, the generated molecules are from the local chemical space around the training molecule. With the help of the RNN-based generators, virtual screening based on local chemical space can be realized which may further improve the traditional models. The proposed idea was evaluated in silico and in practice.

In the virtual part of the evaluation, results of the traditional models and results of the proposed method would be compared through rediscovering abemaciclib as a CDK4/Pim1 dual-target inhibitor. The traditional models were the pharmacophore model prepared for Pim1 and the molecular docking model built for CDK4, which have been prepared as described in part 2.5.

Inactive molecules of both CDK4 and Pim1 collected as described in part 2.5 were screened with the traditional models. Finally, 6 of 150 inactive molecules were wrongly screened, and their ChEMBL IDs were CHEMBL1803075, ChEMBL2443138, CHEMBL1802357, CHEMBL497949, CHEMBL1802355 and CHEMBL3985000. Namely, the traditional models are unable to distinguish abemaciclib from the six inactive molecules.

To obtain the results of virtual screening based on local chemical space, the generator_2 pre-trained with the randomized SMILES of drugs from Drugbank 5.1.2 was used to generate similar molecules of abemaciclib and the six inactive molecules. SMILES sequences of the seven molecules were randomized and randomly mixed. Then, the pre-trained model was trained with the randomized sequences for 30 epochs to convergence, and new sequences were sampled every 10 epochs to generate similar molecules of the seven molecules as many as possible. During each sampling, 200,000 tokens were sampled as described in part 2.2. Repeated and invalid molecules were then removed with RDKit before virtual screening. Unique generated molecules e were then further screened by the pharmacophore model of Pim1 and the molecular docking model of CDK4. After docking, because -PMF was validated as the best scoring function in part 2.5, the first 50% of molecules sorted by their -PMF scores were retained as the results of the proposed method in the first round.

After the first round application of the pipeline, the retained molecules suit the traditional models better. Based on the fact, cluster centers of the retained molecules indicate that the corresponding core structures of the cluster centers are more preferable by the traditional models, so next round of the method was then performed to check the more preferable core structures. In the second round of enumeration and screening for similar molecules, the first screened molecules were clustered into 7 catalogs with the cluster ligand module in DS. The number of clusters parameter was set 7, while the other parameters were set as default so to cluster the molecules according to their Tanimoto distance for their functional connectivity fingerprints with a radius of 3. Then, with the randomized SMILES sequences of the seven cluster centers, the generator trained in the first round was further trained for 20 epochs to convergence. During training, the similar molecules to the seven cluster centers were generated every 10 epochs, and unique molecules were retained. Finally, with the same virtual screening process as described in the first round, the unique molecules were screened, and the screened molecules were the results of the second round.

To test the proposed method in practice, the aim was set to discover new CDK4 inhibitors. The screening was based on the specs_sc_10mg_Apr2019 database (https://www.specs.net/), which is a virtual compound database of molecules collected by Specs (Netherlands), and the compounds in the database are commercially accessible. The database was filtered with the Lipinski and Veber rules, during which no violation was allowed, and molecules with a molecular weight greater than 300 were retained, which corresponds to 105,934 molecules. The molecules were then prepared with the prepare ligands module in DS. The pharmacophore model and the molecular docking model built for CDK4 inhibitors were used to perform virtual screening, and -PMF was used as the scoring function during molecular docking. Because biological tests of all the preliminarily screened molecules could be costly, the first 10 screened molecules sorted by -PMF were molecules whose sequences were randomized. The generator_2 was then trained with the randomized sequences for 30 epochs to convergence, and sampling was performed every 10 epochs. Duplicates were then removed, followed by virtual screening with the pharmacophore model and the molecular docking model built for CDK4. Screened molecules after virtual screening were the final results.

### Origin of compounds through virtual screening

The compounds were obtained from Specs, and their characteristics and purity can be searched at the Specs website (https://www.specs.net/).

### Biological tests on the inhibitory activity of kinases

This service was provided by ChemPartner Co., Ltd, Shanghai, China. (http://www.chempartner.com/) with a mobility shift assay. In tests of CDK4, 10 nM CDK4 (Carna) and 280 μM ATP (Sigma) were added, while 2 nM Pim1 (Carna) and 740 μM ATP were added for tests on Pim1. FAM-P8 (GL Biochem) and FAM-P20 (GL Biochem) at 3 μM were used as substrates for CDK4 and Pim1, respectively. Other details of the tests were as described previously [45]. Staurosporine was tested as a positive control, which is a non-selective inhibitor of kinases.

## Results and discussion

### Generated chemical space with randomized SMILES sequences

**Fig. 2:**
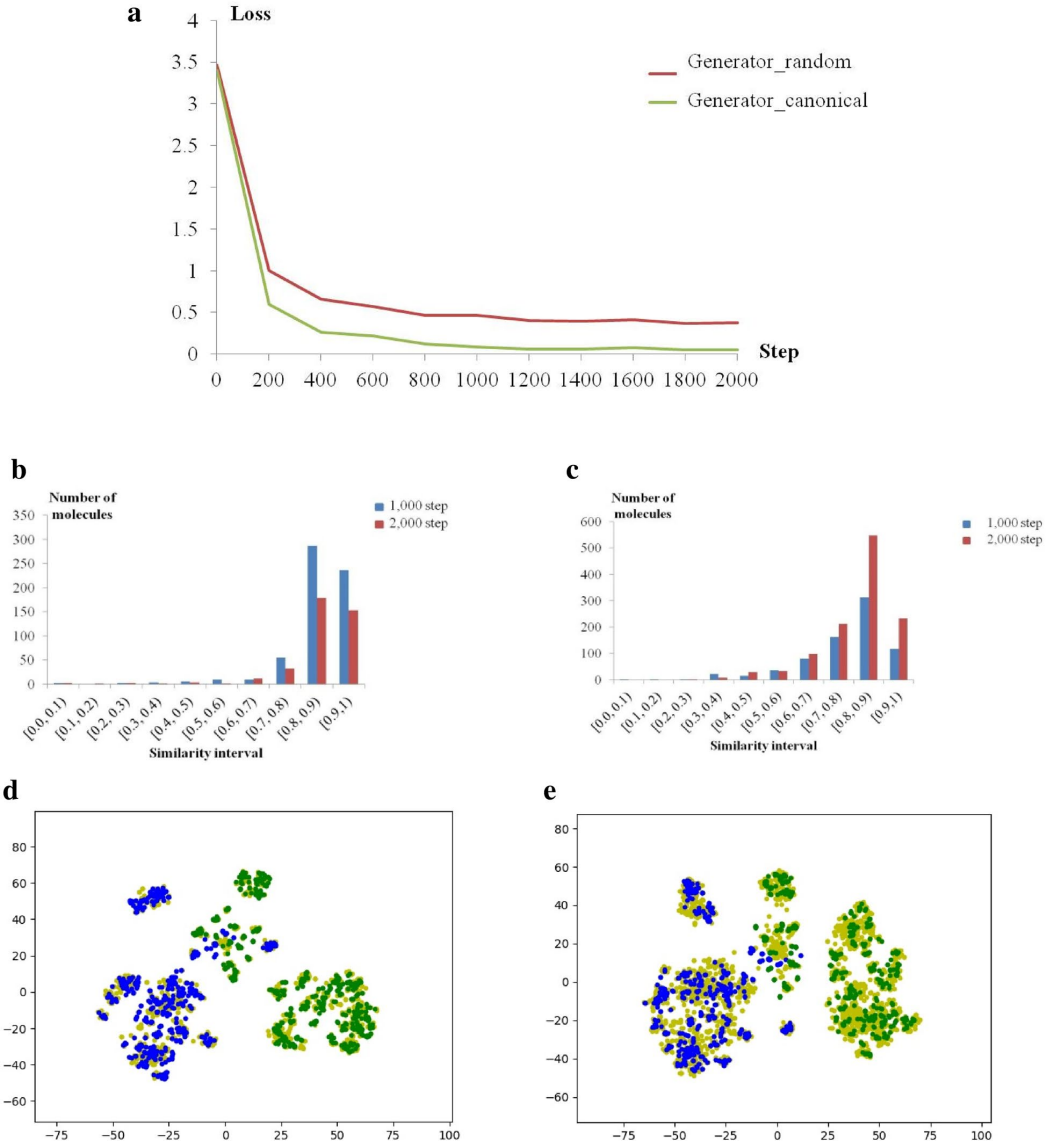
Training and molecule generation based on canonical SMILES sequences and randomized SMILES sequences. **a** The convergence of both models; the loss values were recorded every 200 steps. **b**, **c** Similarity of newly generated unique molecules to their closest inhibitors after training with canonical SMILES sequences (**b**) or randomized SMILES sequences (**c**) for 1000 steps and 2000 steps. **d**, **e** t-SNE plots of combined unique molecules generated through sampling twice after training with canonical SMILES sequences (**d**) or randomized SMILES sequences (**e**). 2-D coordinates of CDK4 inhibitors, Pim1 inhibitors and newly generated molecules are colored blue, green and yellow, respectively

As described previously, TL is often applied to generative models based on RNNs. Because it is hard for models to learn rules from a small quantity of data, TL helps improve molecule generation by pretraining with datasets that are large but not highly related. However, previous models ignore the diversity of SMILES sequences belonging to complex molecules, which helps enlarge datasets so that the enlarged datasets directly related to corresponding tasks can be appropriate inputs. In this study, models trained with or without randomized SMILES sequences were compared at the beginning, corresponding to generator_random and generator_canonical. Both models were trained to convergence (Fig. 2a), and they generated different diversities of their new chemical space. After sampling for 200,000 tokens, the generator_random produces more novel molecules as training continues, while the generator_canonical produces more replicates (Fig. 2b, c). Overall, the generated chemical space around the training molecules was developed better with the dataset of randomized SMILES sequences (Fig. 2d) than with the model trained with canonical SMILES sequences (Fig. 2e). It appears that SMILES randomization can further improve the novelty of generated molecules. In a recent report, it is also found that the randomized SMILES sequences help improve the RNN-based generative models [46]. Notably, SMILES randomization enables a great increase in the number of sequences, which helps solve the problem of data deficiency. TL is frequently used in generative models based on RNNs.

However, generative models trained with TL seem to learn from target molecules after being trained with many unrelated molecules. Though TL brings novelty in terms of the generated molecules, part of the generated molecules may be far from the chemical space of the target molecules, which may require the AI-based models to turn to traditional CADD models. As shown in Fig. 2c, a small number of molecules with a Tanimoto similarity index smaller than 0.6 were generated by the directly trained models so that novelty was partly realized by the model trained directly with the dataset of randomized sequences. In a direct way, randomized SMILES sequences help exploit the chemical space around target molecules. This characteristic may help RNN-based models generate molecules that are novel but similar to known molecules. In regard to the field of medicinal chemistry, reliable, automatic me-too drug design could be realized. Because the chemical space around known active molecules is always protected by strict IP, determining whether the method is practical requires a real application.

### Direct chemical space exploitation around known active molecules

From the chemical space generated by generator_1, three molecules were selected due to their synthetic accessibility. Among the three selected molecules, MI-4 has the lowest similarity index, which indicates its novelty, and both MI-115 and MI-1055 show small modifications based on known inhibitors (Table 1). The originally generated molecules have some fragments that are difficult to attach, so the three molecules were further simplified as MJ-4, MJ-115 and MJ-1055 (Table 1). As the three molecules are similar to known inhibitors, the synthesis of MJ-4, MJ-115 and MJ-1055 is based on the synthesis of similar inhibitors. Compared to the known inhibitors shown in Table 1, MJ-4 has a novel core structure, and both MJ-115 and MJ-1055 introduce hydrophilic fragments into the known inhibitor structures. MJ-4 was synthesized according to Additional file 1: Scheme S1, during which the Buchwald coupling reaction was performed using optimized conditions as previously reported [47]. To avoid the geometric isomerism of the disubstituted cyclohexane, MI-115 was simplified as MJ-115. The novelty of MJ-115 lies in its hydrophilic amide group, and MJ-115 was synthesized according to Additional file 1: Scheme S2. During the simplification of MI-1055, the attachment of the 2,6-difluoro-4-hydroxyphenyl group through the Suzuki coupling reaction was found to be difficult, as mentioned in a recent study [48]. Compared to similar molecules, MJ-1055 retains the novelty of the substituted phenyl group, and MJ-1055 was synthesized according to Additional file 1: Scheme S3. Though the modified molecules were finally obtained, their modifications indicate the potential obstacles that the methods may have, such as the problem of synthetic accessibility.

**Table 1:** Three cases of newly generated molecules and their modifications

Generated molecule	Closest molecule in training set	Tanimoto similarity	Modified molecule
		0.615	
		0.681	
		0.759	

MJ-4 shows weak inhibitory activity on CDK4 (Table 2), which makes MJ-4 a novel lead compound to be further modified and optimized. The pyrrolo[3,2-d]pyrimidine core is a new scaffold compared to that of known CDK4 inhibitors, among which the pyrrolo[2,3-d]pyrimidine fragment is an important fragment. Future modifications will further extend the chemical space around MJ-4, which indicates that there might be ignored space worth fully exploiting even around those explored chemical spaces.

**Table 2:** Inhibitory activity of the three synthesized molecules

Compound	Inhibition for CDK4 at 10 μM (%)	Inhibition for CDK4 at 100 μM (%)	Inhibition for Pim1 at 10 μM (%)
MJ-4	10.96 ± 2.57	72.77 ± 1.24	< 10
MJ-115	35.82 ± 3.75	90.74 ± 1.28	10.38 ± 6.39
MJ-1055	21.06 ± 1.45	81.5 ± 2.2	99.64 ± 0.08

The tests were performed with at least two replicates

Staurosporine was tested as the positive control. Its IC_50_ values for Pim1 and CDK4 are 46 nM and 30 nM, respectively

**Fig. 3:**
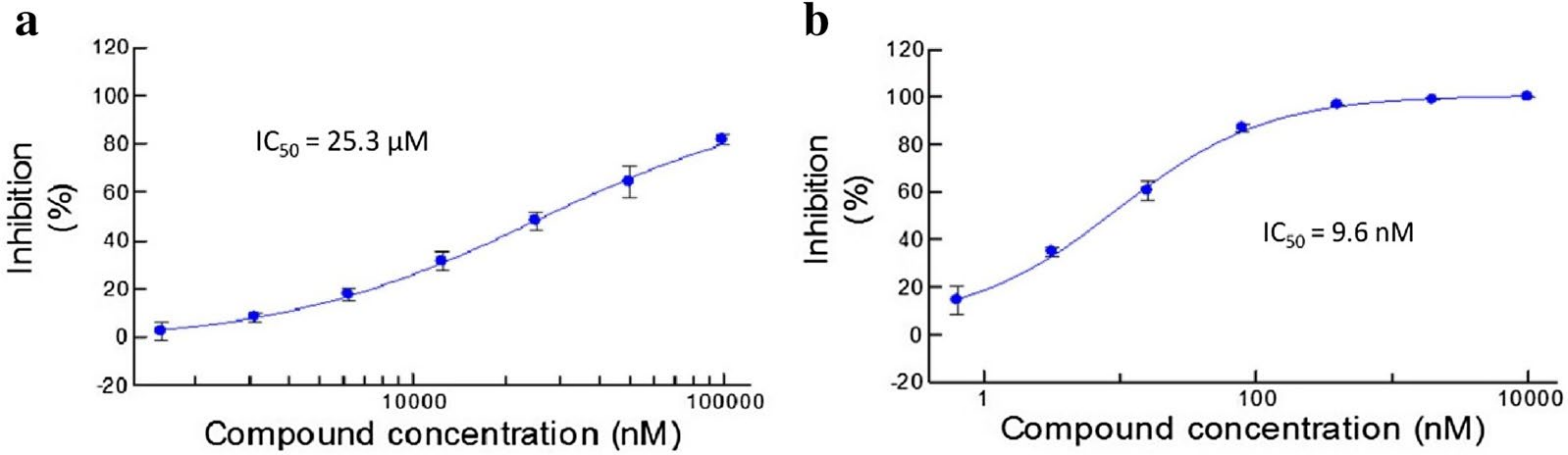
Dose-response curves of MJ-1055 on CDK4 (**a**) and Pim1 (**b**). For each concentration, tests were performed with at least two replicates

The activity of MJ-115 was obviously reduced (Table 2) when compared to that of its closest inhibitor in the training molecules. This disappointing result implies that the RNN-based generative model still needs help from accurate models designed for virtual screening because the generated molecules may not maintain desired activity. Luckily, in the case of MJ-1055, AI-based reorganization could help discover treasures around the space explored previously. MJ-1055 retains potent inhibitory activity on Pim1 by the attachment hydroxyl groups to the hydrophobic phenyl groups, and the IC_50_ of MJ-1055 is 9.6 nM, as shown in Fig. 3a. Perhaps the model found that the hydroxyl-substituted phenyl moiety is still an active fragment among the training molecules, so the introduction of the group successfully maintains potent activity. Notably, because the phenyl moiety is considered a hydrophobic pharmacophore, the attachment of the hydrophilic hydroxyl group makes MJ-1055 different from similar molecules protected in a relevant Markush patent [41]. MJ-1055 also shows weak inhibitory activity on CDK4, with an IC_50_ of 25.3 μM (Fig. 3b), which supports the applicability and potential of RNN-based generative models in real tasks. Although not all the molecules retain high potency, they can still be defined as inhibitors with weak or strong inhibitory activity. Even if the chemical space around the training molecules has been explored, RNN-based models trained with randomized SMILES sequences help make full use of the space, which would further improve the efficiency and accuracy of drug discovery.

### Virtual screening from the perspective of local chemical space

The cases mentioned above suggest that the chemical space of known inhibitors could be local. The idea that the chemical space of active molecules could be local is also supported by SARs, which are commonly used by medicinal chemists. In many cases, small modifications may affect the activity levels but not the property of being active molecules. With this belief, the hypothesis seems reasonable that, compared to a single molecule being screened out, a group of screened molecules which are generated based on the single molecule indicate that the single molecule is more likely to be active.

The best pharmacophore model of Pim1 was built from a complex structure with the PDB code 5TUR, and its specificity, sensitivity and AUC were 0.79, 0.87 and 0.806, respectively. The best pharmacophore model of CDK4 was built from a complex structure belonging to CDK6 (PDB code: 3NUP), whose specificity, sensitivity and AUC are 0.61, 0.84 and 0.73, respectively.

The performance of the pharmacophore model for CDK4 is just acceptable, so an additional molecular docking model for CDK4 was prepared. LigandFit achieves a specificity of 0.53 and a sensitivity of 0.85. The specificity is still unsatisfactory, which means that LigandFit could easily be confounded by inactive molecules. Scoring functions were then compared through receiver operating characteristic (ROC) curves (Additional file 1: Figure S1), and -PMF achieves the highest AUC of 0.821 (Additional file 1: Table S1), which indicates that a higher -PMF score yields a larger probability of discovering a CDK4 inhibitor.

The proposed idea was first compared to traditional virtual screening methods in silico. Abemaciclib shows potent inhibitory activity on both CDK4 and Pim1. With the pharmacophore model built for Pim1 and the molecular docking model based on CDK4, not only abemaciclib was screened, but another six inactive molecules were wrongly screened as well, and they cannot even be distinguished with -PMF (Table 3), which has been validated as an effective scoring function. Namely, the traditional models are unable to distinguish abemaciclib from the six inactive molecules.

**Table 3:** Virtual screening results of the six inactive molecules and abemaciclib

Molecules	Pharmacophore model for Pim1	Molecular docking model for CDK4	-PMF
Abemaciclib	√	√	95.92
ChEMBL1803075	√	√	85.1
ChEMBL2443138	√	√	113.57
ChEMBL1802357	√	√	105.94
ChEMBL497949	√	√	91.07
ChEMBL1802355	√	√	93.5
ChEMBL3985000	√	√	85.91

**Fig. 4:**
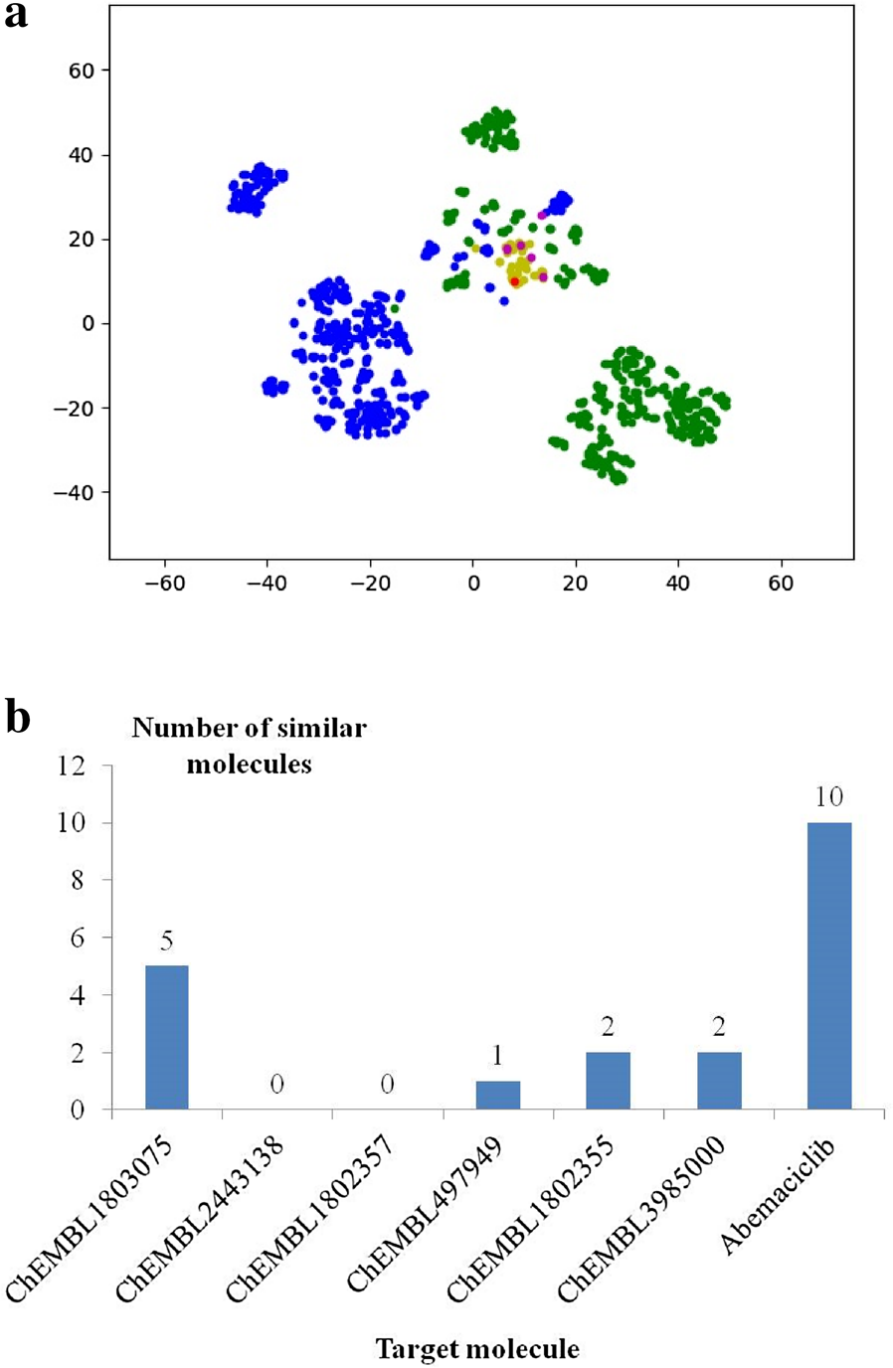
The filtered molecules after the first round of virtual screening with the pharmacophore model for Pim1 and the molecular docking model for CDK4 and -PMF. **a** t-SNE plot of CDK4 inhibitors (blue), Pim1 inhibitors (green), abemaciclib (red), inactive molecules (magenta) and screened molecules (yellow). **b** Number of the most similar molecules compared to the seven target molecules

With a trained RNN-based generator, instead of seven individual molecules, objects of the traditional models are seven groups of molecules similar to the seven molecules. The pre-trained generator_2 was trained with randomized sequences belonging to the seven molecules, and the similar molecules to the seven molecules were successfully obtained (Additional file 1: Figure S2) through sampling during training. After virtual screening for the potential active molecules, molecules that are most similar to abemaciclib were kept at most, which is shown with t-SNE (Fig. 4a) and similarity analysis (Fig. 4b).

**Fig. 5:**
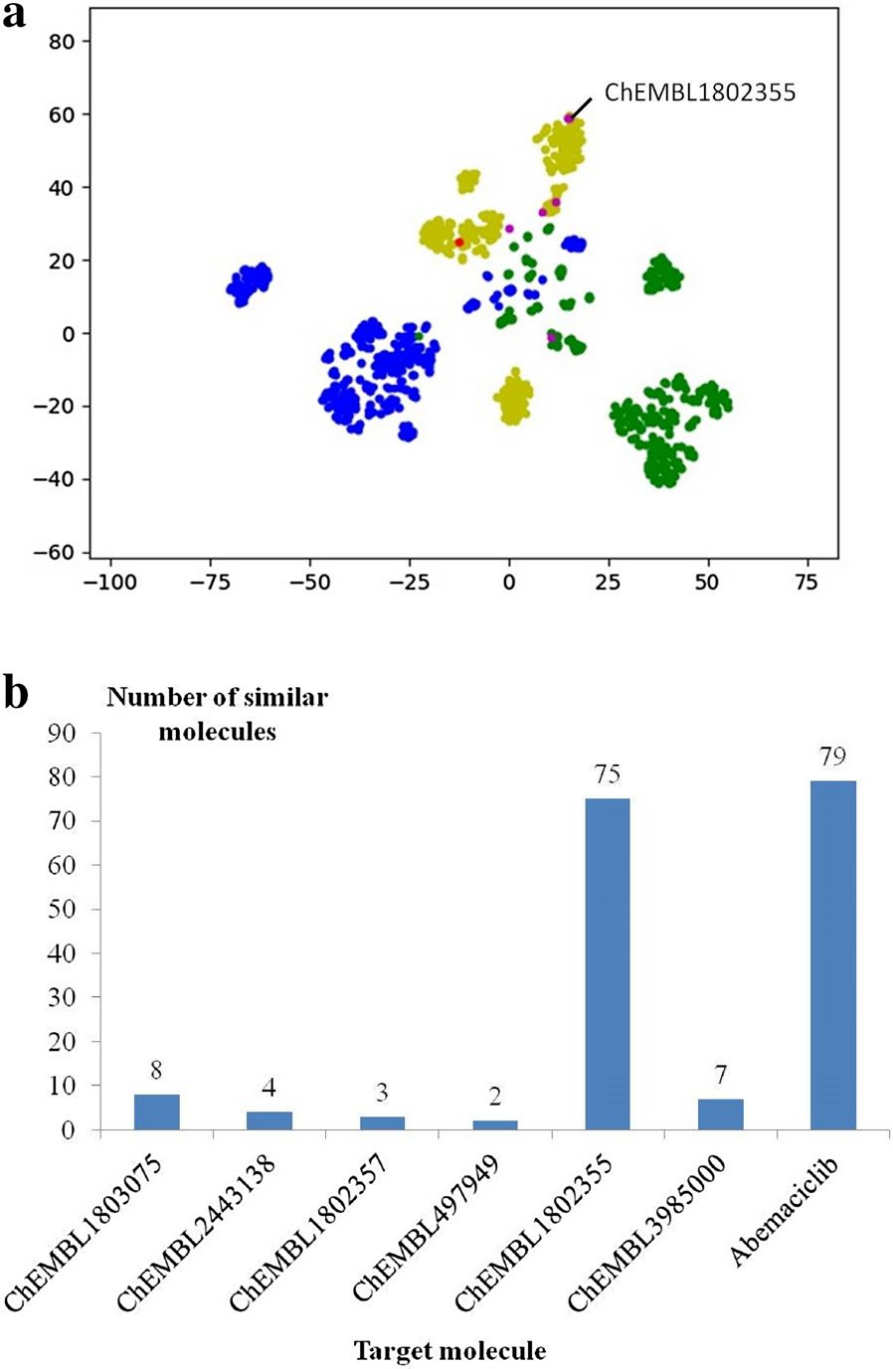
The filtered chemical space after the second round of virtual screening with the pharmacophore model for Pim1 and the molecular docking model for CDK4 and -PMF. **a** t-SNE plot of CDK4 inhibitors (blue), Pim1 inhibitors (green), abemaciclib (red), inactive molecules (magenta) and screened molecules (yellow). **b** Number of the most similar molecules compared to the seven target molecules

**Fig. 6:**
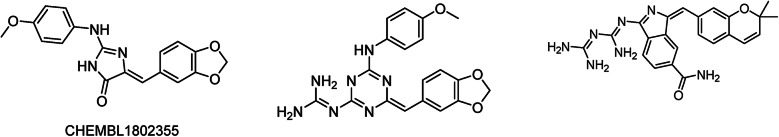
Representative molecules in the transferred chemical space moving away from CHEMBL1802355. Molecules from left to right are CHEMBL1802355, the most similar molecule of CHEMBL1802355 and the cluster center of the chemical space to which CHEMBL1802355 belongs

After the first cycle of generating similar molecules and enrichment with virtual screening, the first 50% of screened molecules sorted by their -PMF score were kept as the potential active molecules. Compared to the preliminary generated molecules, the screened molecules meet the requirements of the virtual screening models better, which represent more ideal molecules with preferable structures. To find the core structures shared in the screened molecules, those molecules were represented by seven centers through clustering according to their structures, and the seven cluster centers represent more preferable core structures for the virtual screening models compared to the structures of the original seven molecules. Then, the second cycle of molecule generation and virtual screening was performed to discover structures that are more preferable for the virtual screening models. With t-SNE (Fig. 5a) and similarity analysis (Fig. 5b), the space around abemaciclib was further emphasized as expected, while the local space near CHEMBL1802355 was also enriched. From the result of t-SNE (Fig. 5a), the screened molecules appear to be distant from CHEMBL1802355. Then, the closest molecule of CHEMBL1802355 based on Tanimoto similarity and the cluster center of the screened space around CHEMBL1802355 were found to check the new preferable structure for the virtual screening models. As shown in Fig. 6, both molecules possess extra guanidyl groups, and it is the same for most generated molecules in the local space around ChEMBL1802355. As guanidyl is a basic group, it conforms well to the positive pharmacophore in the model of Pim1 (Additional file 1: Figure S3), and the basic fragment matches well with the SAR reported in a study on Pim1 inhibitors [49], where ChEMBL1802355 was found to lack activity and the introduction of basic fragments helps improve the inhibitory activity.

**Fig. 7:**
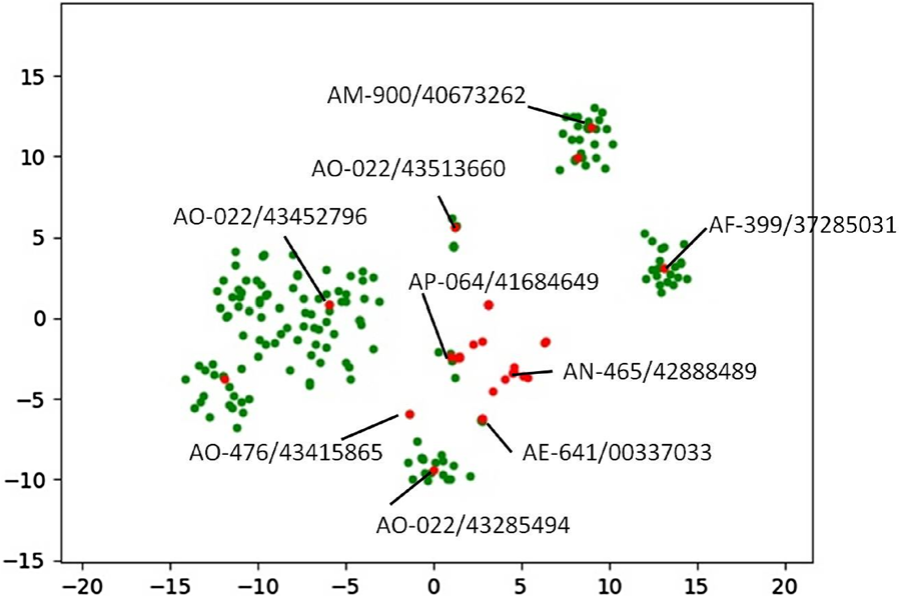
t-SNE plot of the screened molecules. Coordinates of molecules directly screened by the pharmacophore model and the molecular docking model built for CDK4 are labeled in red. After chemical space extension of 10 molecules with higher -PMF scores and the second round of virtual screening, the molecules whose coordinates are in green were finally screened. The compounds finally obtained and tested were flagged with their ID number

The hypothesis was then further supported with a real test. The aim was preliminarily set to discover new inhibitors of CDK4. The pharmacophore model built for CDK4 and the molecular docking model aided by -PMF were used to perform virtual screening. Potential active molecules were first screened out. After one cycle of molecule generation based on the preliminarily screened molecules and virtual screening, the screened preferable molecules for the virtual screening models were visualized with t-SNE (Fig. 7), and molecules (flagged in Fig. 7) from the large local space and small local space were obtained and tested. Among those molecules, only AF-399/37285031 is found as a new CDK4 inhibitor, with inhibition of 57.8 ± 5.0% at 10 μM, which proves the reliability of this method in practice. As shown in Fig. 7, the chemical space around AF-399/37285031 is retained through virtual screening, and the chemical space around several inactive molecules shown in Fig. 7 fails to be enriched. However, not all the molecules selected from the enriched chemical space show activity. The reason may be due to unreliable traditional models. Although those traditional models may be inaccurate, virtual screening from the perspective of groups of similar molecules still help improve the accuracy.

## Conclusions

Among AI-based generative models, though powered by the simplest algorithm, models based on RNNs have proven their potential in de novo molecular generation. Although these models perform reorganization of sequences without optimization, key fragments contributing to the activity can be effectively extracted and further assembled to afford novel and potentially active generated molecules. More importantly, an ignored detail that a complex molecule may have different SMILES sequences helps effectively generate molecules that are similar to training molecules, and the characteristic further improves the models’ ability to exploit the chemical space around those training molecules, which may be helpful for the industry to solve IP issues. However, the models can still be further improved. During the application of the models, synthetic accessibility and stability exhibited problems, perplexing us, which could represent the obstacles for AI-based generative models.

To explore new chemical spaces of active molecules, RNN-based models show their applicability. With vivid cases reported in this study, traditional virtual screening could be further improved from the perspective of local chemical space. On the one hand, the accuracy of virtual screening may be further improved. On the other hand, the cycle of molecule generation and virtual screening could guide discovery of ideal structures that match traditional models much better, which would help guide further structural modifications. In regard to new targets for which there is little knowledge of active molecules, virtual screening based on local chemical space shows priority when compared to traditional models that could be inaccurate without validation and to AI models desperate for abundant data.

Overall, RNNs deal with the raw representation of molecules well, and this feature makes RNNs good at exploring the chemical space. With further improvement and application of the models, AI may realize more efficient and accurate drug discovery and ultimately bolster more fierce competition for the industry in the future.

## Supplementary information

**Additional file 1.** Additional information on virtual screening and compound synthesis.

## Data Availability

All data and scripts to build the models are provided at https://github.com/Xyqii/RNN_generator.

## Abbreviations

R&D: Research and development
ML: Machine learning
AI: Artificial intelligence
CDK4: Cyclin-dependent kinase 4
DL: Deep learning
NNs: Neural networks
RNNs: Recurrent neural networks
SMILES: Simplified molecular input line entry specification
InChI: International chemical identifier
TL: Transfer learning
IP: Intellectual property
Pim1: Proviral integration site for Moloney murine leukemia virus kinase 1
DS: Discovery studio
LSTM: Long short-term memory
t-SNE: t-distributed stochastic neighbor embedding
