# Supplementary material for: Chemical space exploration based on recurrent neural networks: applications in discovering kinase inhibitors
Source: J Cheminform. 2020 Jun 8;12:42. doi: 10.1186/s13321-020-00446-3 (PMC7278228; doi:10.1186/s13321-020-00446-3)
Supplement: Supplementary file 1 — Additional file 1. Additional information on virtual screening and compound synthesis. [file 13321_2020_446_MOESM1_ESM.docx]

Additional file

Chemical Space Exploration Based on Recurrent Neural Networks: Applications in Discovering Kinase Inhibitors

Xuanyi Li^1^, Yinqiu Xu^1^, Hequan Yao^**, 1^, Kejiang Lin^*, 1^

*^1^Department of Medicinal Chemistry, School of Pharmacy, China Pharmaceutical University, Nanjing 210009, China*

*Author for correspondence: link@cpu.edu.cn

**Author for correspondence: hyao@cpu.edu.cn

**Contents:**

1. Table S1. The AUC values of ROC curves belonging to different scoring functions......................................................................................S2

2. Figure S1. ROC curves of different scoring functions........................S3

3. Figure S2. The extended chemical space around the seven molecules used for validation *in silico*......................................................................S4

4. Figure S3. The best pharmacophore model built for Pim1.................S6

5. Scheme S1. Synthesis of MJ-4.............................................................S7

6. Scheme S2. Synthesis of MJ-115.........................................................S8

7. Scheme S3. Synthesis of MJ-1055.......................................................S9

8. Synthesis............................................................................................S10

9. ^1^H NMR spectra.................................................................................S20

10. ^13^C NMR spectra..............................................................................S26

References..............................................................................................S32

Table S1. The AUC values of ROC curvesbelonging to different scoring functions.

| Scoring function | AUC |
| --- | --- |
| -PLP1 | 0.437 |
| -PLP2 | 0.473 |
| -PMF | 0.821 |
| -PMF04 | 0.762 |
| DOCK_SCORE | 0.541 |
| Jain | 0.672 |
| LigScore1_Dreiding | 0.371 |
| LigScore2_Dreiding | 0.374 |
| Ludi_1 | 0.575 |
| Ludi_2 | 0.595 |
| Ludi_3 | 0.738 |


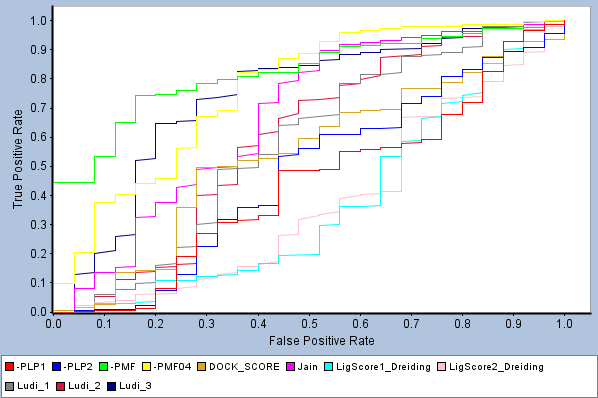


Figure S1. ROC curves of different scoring functions. The highest score of different poses belonging to each molecule was used to obtain the curves.

A


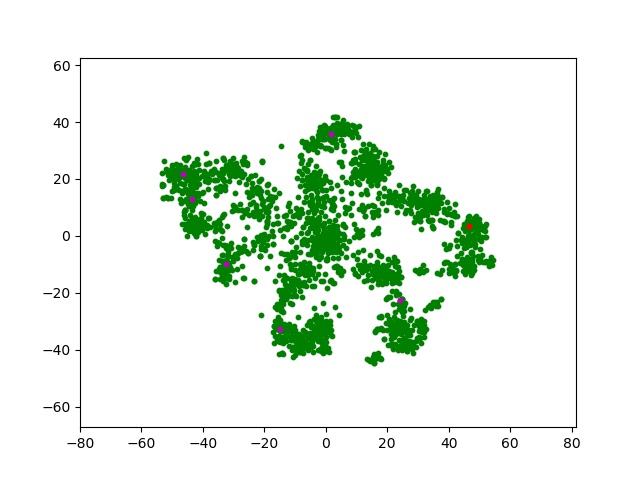


B


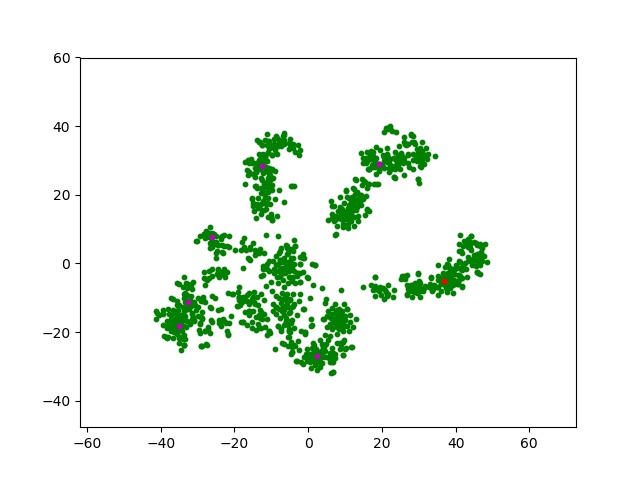


C


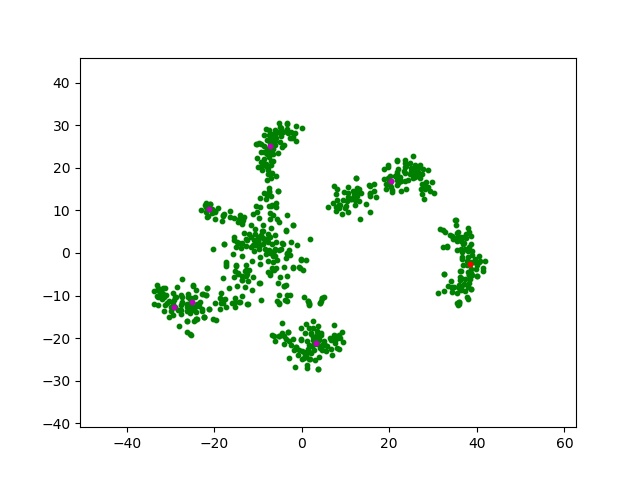


Figure S2. The extended chemical space around the seven molecules used for validation in silico. The molecules were sampled after 5 epochs (A), 10 epochs (B) and 15 epochs (C) of TL. Unique molecules were kept to show the newly generated chemical space. Abemaciclib, inactive molecules and generated molecules are colored red, magenta and green, respectively.


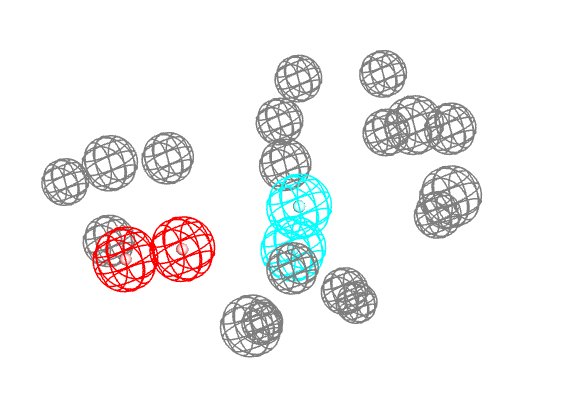


Figure S3. The best pharmacophore model built for Pim1. Ionizable groups with positive charges, hydrophobic groups and excluded volumes are shown in red, blue and gray, respectively.

Scheme S1. Synthesis of MJ-4^a^.

^a^Reagents and conditions: (a) CuI, K_3_PO_4_, DMF, 110 °C; (b) (t-Bu_3_P)_2_Pd, K_3_PO_4_, DMF, 110 °C; (c) 4 M HCl in EA.

Scheme S2. Synthesis of MJ-115^a^.

^a^Reagents and conditions: (a) Cs_2_CO_3_, DMF; (b) i-PrMgCl. LiCl, TosCN, THF, -78 °C; (c) (t-Bu_3_P)_2_Pd, K_3_PO_4_, DMF, 110 °C; (d) K_2_CO_3_, 30% H_2_O_2_, DMSO; (e) 4 M HCl in EA.

Scheme S3. Synthesis of MJ-1055 ^a^.

 ^a^Reagents and conditions: (a) Cs_2_CO_3_, DMF, 100 °C; (b) Pd(PPh_3_)_4_, Na_2_CO_3_, 1,4-dioxane/H_2_O(4:1), 100 °C; (c) 4 M HCl in EtOH.

**8. Synthesis**

**General Chemistry.** All commercial starting materials and solvents were reagent grade and were used without further purification unless otherwise noted. PE refers to petroleum ether, b.p. 60 − 90 °C, and EA refers to ethyl acetate. ^1^H NMR and ^13^C NMR spectra were recorded on a Bruker AV-300 (300 MHz) or Bruker AV-400 (400 MHz) spectrometer. Chemical shifts (δ) were reported in parts per million (ppm) using tetramethylsilane as an internal standard. Coupling constants (J) are expressed in Hz, and spin multiplicities are given as s (singlet), d (doublet), t (triplet), q (quartet), dd (doublet of doublets), dt (doublet of triplets), m (multiplet) and br (broad). High-resolution mass spectrometry (HRMS) was performed on an Agilent 6530 Q-TOF mass spectrometer with electron spray ionization (ESI) as the ion source. TLC was performed on Huanghai HSGF 254 silica gel plates (Yantai, China). Silica gel (200-300 mesh) manufactured by Qingdao Haiyang Chemical Group Co., Ltd. (Qingdao, China) was used for chromatography. All bioassayed compounds obtained through synthesis were purified to ≥95% purity as determined by a Shimadzu high-performance liquid chromatography (HPLC) system equipped with a C18 column and a UV detector using the following method: detection wavelength, 254 nm; mobile phase, MeOH/H_2_O containing 1‰ CF_3_COOH (50:50) or MeOH containing 1‰ CF_3_COOH /H_2_O (80:20); and flow rate, 1.0 mL/min.

*2-Chloro-5-(3-methoxyphenyl)-5H-pyrrolo[3,2-d]pyrimidine* ***(1)***. To a Schlenk tube was added 2-chloro-5H-pyrrolo[3,2-d]pyrimidine (30.7 mg, 0.2 mmol), CuI (3.81 mg, 0.02 mmol), K_3_PO_4_ (84.9 mg, 0.4 mmol) and a stir bar, and the tube was evacuated and backfilled with Ar three times. A solution of 1-iodo-3-methoxybenzene (28.6 μL, 0.24 mmol) and N,N'-dimethyl-1,2-cyclohexanediamine (12.6 μL, 0.08 mmol) in 1 mL of anhydrous DMF was then added to the tube. The tube was sealed and stirred for 16 h at 110 °C. The reaction mixture was filtered through Celite and concentrated under vacuum. The product was purified by chromatography (0-9% EA/PE) to afford the desired product 1 as a white solid (51.4 mg, yield 99%). ^1^H NMR (300 MHz, chloroform-*d*) δ 8.84 (d, *J* = 0.8 Hz, 1H), 7.77 (d, *J* = 3.3 Hz, 1H), 7.49 (td, *J* = 8.0, 0.7 Hz, 1H), 7.14 – 6.92 (m, 3H), 6.81 (dd, *J* = 3.3, 0.8 Hz, 1H), 3.89 (s, 3H) ppm; ^13^C NMR (75 MHz, chloroform-*d*) δ 163.6, 156.6, 155.8, 144.0, 141.3, 138.8, 133.7, 129.1, 118.4, 116.1, 112.6, 106.2, 58.2 ppm; HRMS (ESI) calcd for [C_13_H_10_ClN_3_O+H]^+^ 260.0585, found 260.0584.

*tert-Butyl-4-(4-((5-(3-methoxyphenyl)-5H-pyrrolo[3,2-d]pyrimidin-2-yl)amino)phenyl)piperazine-1-carboxylate* ***(2)***. To a Schlenk tube was added **1** (23.5 mg, 0.1 mmol), 1-Boc-4-(4-aminophenyl)piperazine (33.3 mg, 0.12 mmol), (t-Bu_3_P)_2_Pd (2.5 mg, 5 mol %), K_3_PO_4_ (42.4 mg, 0.2 mmol) and a stir bar, and the tube was evacuated and backfilled with Ar three times. Then, 1 mL of anhydrous DMF was added to the tube under an Ar atmosphere. The tube was sealed and stirred for 24 h at 110 °C. The reaction mixture was filtered through Celite and concentrated under vacuum. The product was purified by chromatography (0-20% EA/PE) to afford the desired product **2** as a pale yellow solid (29 mg, yield 57.9%). ^1^H NMR (300 MHz, chloroform-*d*) δ 8.72 (s, 1H), 7.62 – 7.54 (m, 3H), 7.44 (t, *J* = 8.1 Hz, 1H), 7.04 (d, *J* = 8.0 Hz, 1H), 7.00 – 6.90 (m, 5H), 6.58 (d, *J* = 3.2 Hz, 1H), 3.88 (s, 3H), 3.62 – 3.57 (m, 4H), 3.10 – 3.02 (m, 4H), 1.49 (s, 9H) ppm; ^13^C NMR (75 MHz, chloroform-*d*) δ 160.9, 156.1, 154.8, 153.2, 146.6, 141.0, 139.6, 134.1, 134.0, 130.9, 123.3, 120.4, 118.0, 115.3, 112.4, 109.2, 102.7, 79.8, 55.6, 50.6, 28.5 ppm; HRMS (ESI) calcd for [C_28_H_32_N_6_O_3_+H]^+^ 501.2609, found 501.2601.

*5-(3-Methoxyphenyl)-N-(4-(piperazin-1-yl)phenyl)-5H-pyrrolo[3,2-d]pyrimidin-2-amine* ***(MJ-4)*** *hydrochloride*. A mixture of **2** (29 mg, 0.058 mmol) and 4 M HCl in EA (1 mL) was stirred at room temperature for 30 min. The mixture was then filtered, and the residue was washed with EA to afford the desired product MJ-4 as a pale brown solid (22.8 mg, yield 90.1%). ^1^H NMR (400 MHz, CD_3_OD) δ 8.92 (d, *J* = 4.3 Hz, 1H), 8.26 (d, *J* = 3.6 Hz, 1H), 7.62 – 7.41 (m, 3H), 7.36 – 7.00 (m, 6H), 6.69 (d, *J* = 3.5 Hz, 1H), 3.90 (d, *J* = 3.6 Hz, 3H), 3.59 – 3.49 (m, 4H), 3.48 – 3.39 (m, 4H) ppm; ^1^H NMR (300 MHz, DMSO) δ 9.56 (s, 2H), 9.20 (s, 1H), 8.84 (s, 1H), 8.09 (s, 1H), 7.75 (d, *J* = 8.4 Hz, 2H), 7.49 (t, *J* = 8.1 Hz, 1H), 7.23 (d, *J* = 12.8 Hz, 2H), 6.97 (t, *J* = 8.5 Hz, 3H), 6.59 (d, *J* = 3.1 Hz, 1H), 3.87 (s, 3H), 3.30 (s, 4H), 3.20 (s, 4H) ppm; ^13^C NMR (75 MHz, CD_3_OD) δ 160.3, 150.6, 150.3, 145.1, 141.0, 139.6, 137.9, 132.0, 131.0, 121.6, 117.3, 115.3, 113.7, 108.9, 101.3, 55.7, 46.4, 42.3 ppm; HRMS (ESI) calcd for [C_23_H_24_N_6_O+H]^+^ 401.2084, found 401.2082. HPLC analysis: MeOH/H_2_O containing 1‰ CF_3_COOH (50:50), 6.42 min, 97.9% purity.

*2-Chloro-5-iodo-7-isopropyl-7H-pyrrolo[2,3-d]pyrimidine* ***(3)***. 2-Chloro-5-iodo-7H-pyrrolo[2,3-d] pyrimidine (139.7 mg, 0.5 mmol), 2-iodopropane (75 μL, 0.75 mmol) and Cs_2_CO_3_ (244.4 mg, 0.75 mmol) were added to 2 mL of DMF, and the mixture was stirred for 3 h at room temperature. The reaction was quenched by the addition of water (10 mL) and extracted with EA (3 × 10 mL), and the organic layers were separated and combined. The combined organic layer was washed with brine, dried over anhydrous sodium sulfate, and concentrated under vacuum. The product was purified by chromatography (10% EA/PE) to afford the desired product **3** as a white solid (140 mg, yield 87.1%). ^1^H NMR (300 MHz, chloroform-*d*) δ 8.56 (s, 1H), 7.42 (s, 1H), 5.12 (p, *J* = 6.8 Hz, 1H), 1.53 (d, *J* = 6.8 Hz, 6H) ppm; ^13^C NMR (75 MHz, chloroform-*d*) δ 154.3, 151.7, 151.5, 130.6, 120.4, 52.5, 46.8, 22.8 ppm; HRMS (ESI) calcd for [C_9_H_9_ClIN_3_+H]^+^ 321.9602, found 321.9601.

*2-Chloro-7-isopropyl-7H-pyrrolo[2,3-d]pyrimidine-5-carbonitrile* ***(4)***. First, 1.3 M i-PrMgCl. LiCl in THF (461.5 μL, 0.6 mmol) was added to anhydrous THF (2.50 mL) at -78 °C under Ar. Compound **3** (64.3 mg, 0.2 mmol) in THF (2 mL) was added dropwise, and the reaction was stirred at -78 °C for 30 min. Then, tosyl cyanide (79.7 mg, 0.44 mmol) in THF (2 mL) was added, and the reaction was stirred at -78 °C for another 30 min. HOAc (0.1 mL) was added to the solution and stirred at room temperature for 30 min, and then, water (10 mL) was added, and the solution was and extracted with EA (3 × 10 mL). The combined organic layer was washed with brine, dried over anhydrous sodium sulfate, and concentrated under reduced pressure. The product was purified by chromatography (2-5% EA/PE) to give **4** as a white solid (19 mg, yield 43.1%). ^1^H NMR (300 MHz, chloroform-*d*) δ 8.99 (s, 1H), 7.87 (s, 1H), 5.16 (hept, *J* = 6.7 Hz, 1H), 1.59 (d, *J* = 6.8 Hz, 6H) ppm; ^13^C NMR (75 MHz, chloroform-*d*) δ 155.5, 151.2, 151.1, 134.0, 117.1, 113.3, 85.4, 47.9, 22.5 ppm; HRMS (ESI) calcd for [C_10_H_9_ClN_4_+H]^+^ 221.0589, found 221.0588.

*tert-Butyl-4-(4-((5-cyano-7-isopropyl-7H-pyrrolo[2,3-d]pyrimidin-2-yl)amino)phenyl)piperazine-1-carboxylate* ***(5)***. To a Schlenk tube was added **4** (22.1 mg, 0.1 mmol), 1-Boc-4-(4-aminophenyl)piperazine (27.7 mg, 0.1 mmol), (t-Bu_3_P)_2_Pd (5 mg, 10 mol %), K_3_PO_4_ (42.4 mg, 0.2 mmol) and a stir bar, and the tube was evacuated and backfilled with Ar three times. Then, 1 mL of anhydrous DMF was added to the tube under an Ar atmosphere. The tube was sealed and stirred for 24 h at 110 °C. The reaction mixture was filtered through Celite and concentrated under vacuum. The product was purified by chromatography (PE; 0-2% MeOH/DCM) to afford the desired product **5** as a pale yellow solid (21.3 mg, yield 46.2%). ^1^H NMR (400 MHz, chloroform-*d*) δ 8.74 (s, 1H), 7.78 (s, 1H), 7.59 (d, *J* = 9.0 Hz, 2H), 7.54 (s, 1H), 6.97 (d, *J* = 9.0 Hz, 2H), 5.02 – 4.91 (m, 1H), 3.63 – 3.55 (m, 4H), 3.14 – 3.06 (m, 4H), 1.54 (d, *J* = 6.8 Hz, 6H), 1.50 (s, 9H) ppm; ^13^C NMR (101 MHz, chloroform-*d*) δ 157.3, 154.8, 151.4, 150.6, 146.9, 133.0, 130.6, 120.6, 117.6, 114.8, 111.4, 84.7, 79.9, 50.2, 47.0, 28.5, 22.4 ppm; HRMS (ESI) calcd for [C_25_H_31_N_7_O_2_+H]^+^ 462.2612, found 462.2610.

*tert-Butyl-4-(4-((5-carbamoyl-7-isopropyl-7H-pyrrolo[2,3-d]pyrimidin-2-yl)amino)phenyl)piperazine-1-carboxylate* ***(6)***. To a mixture of **5** (74.5 mg, 0.16 mmol) in 3 mL of DMSO, cooled in an ice bath, were added K_2_CO_3_ (50 mg) and 30 wt% H_2_O_2_. The mixture was stirred at room temperature for 1 h. Then, 10 mL of water was added to the mixture, and the precipitate was filtered off. The product was purified by chromatography (EA) to afford the desired product **6** as a pale yellow solid (56.3 mg, yield 73.4%). ^1^H NMR (300 MHz, chloroform-*d*) δ 9.01 (s, 1H), 7.67 – 7.55 (m, 4H), 7.03 – 6.88 (m, 2H), 6.08 (s, 2H), 5.07 – 4.88 (m, 1H), 3.63 – 3.56 (m, 4H), 3.08 (t, *J* = 5.2 Hz, 4H), 1.54 (s, 3H), 1.52 (s, 3H), 1.49 (s, 9H) ppm; ^13^C NMR (75 MHz, DMSO-*d*_6_) δ 165.5, 156.6, 154.3, 151.9, 145.9, 134.6, 126.2, 119.7, 117.3, 110.9, 110.4, 79.4, 55.4, 50.0, 46.2, 28.5, 22.6 ppm; HRMS (ESI) calcd for [C_25_H_33_N_7_O_3_+H]^+^ 480.2718, found 480.2714.

*7-Isopropyl-2-((4-(piperazin-1-yl)phenyl)amino)-7H-pyrrolo[2,3-d]pyrimidine-5-carboxamide* ***(MJ-115)*** *hydrochloride*. A mixture of **6** (56.3 mg, 0.117 mmol) and 4 M HCl in EA (2 mL) was stirred at room temperature for 30 min. The mixture was then filtered, and the residue was washed with EA and DCM to afford the desired product MJ-115 as a white solid (38.7 mg, yield 79.5%). ^1^H NMR (400 MHz, DMSO-*d*_6_) δ 9.91 (s, 1H), 9.68 (s, 2H), 9.00 (s, 1H), 8.39 (s, 1H), 7.81 (s, 1H), 7.66 (s, 2H), 7.12 (s, 1H), 7.00 (s, 2H), 4.83 (s, 1H), 3.36 (s, 4H), 3.16 (d, *J* = 15.9 Hz, 4H), 1.46 (s, 6H) ppm; ^13^C NMR (101 MHz, DMSO-*d*_6_) δ 165.0, 154.0, 152.4, 148.3, 145.5, 133.4, 128.9, 120.7, 117.4, 111.1, 111.0, 46.7, 46.7, 43.0, 22.6 ppm; HRMS (ESI) calcd for [C_20_H_25_N_7_O+H]+ 380.2193, found 380.2190. HPLC analysis: MeOH/H_2_O containing 1‰ CF_3_COOH (50:50), 3.07 min, 96.8% purity.

*2-(6-Bromo-[1,2,4]triazolo[4,3-a]pyridin-3-yl)quinolin-8-ol* ***(7)***. Compound **7** was prepared as described previously [1]. The product was an off-white solid. ^1^H NMR (300 MHz, DMSO-*d*_6_) δ 10.51 (s, 1H), 10.43 (s, 1H), 8.45 (q, *J* = 8.7 Hz, 2H), 7.97 (d, *J* = 9.6 Hz, 1H), 7.69 (dd, *J* = 9.7, 1.8 Hz, 1H), 7.54 – 7.37 (m, 2H), 7.20 (dd, *J* = 7.1, 2.0 Hz, 1H) ppm; ^13^C NMR (75 MHz, DMSO-*d*_6_) δ 154.1, 149.8, 145.8, 138.2, 137.7, 132.3, 129.0, 128.7, 128.1, 119.7, 118.4, 116.9, 113.5, 109.8 ppm; HRMS (ESI) calcd for [C_15_H_9_BrN_4_O+H]^+^ 341.0038, found 341.0038.

*tert-Butyl-4-((2-(6-bromo-[1,2,4]triazolo[4,3-a]pyridin-3-yl)quinolin-8-yl)oxy)piperidine-1-carboxylate* ***(8)***. A mixture of **7** (68.2 mg, 0.2 mmol), 1-Boc-4-methanesulfonyloxypiperidine (83.7 mg, 0.3 mmol), Cs_2_CO_3_ (195.5 mg, 0.6 mmol) and DMF (2 mL) was stirred at 100 °C overnight. The reaction was then cooled to room temperature, washed with water (20 mL) and extracted with EA (3 × 10 mL). The combined organic layers were washed with brine, dried over Na_2_SO_4_, filtered and concentrated in vacuum. The crude product was purified by flash chromatography on silica gel (1-30% EA/PE) to afford the desired product **8** as a white foam (118.7 mg, yield 19%). ^1^H NMR (300 MHz, chloroform-*d*) δ 10.61 (s, 1H), 8.62 (d, *J* = 8.5 Hz, 1H), 8.29 (d, *J* = 8.7 Hz, 1H), 7.82 (d, *J* = 9.6 Hz, 1H), 7.50 (dd, *J* = 15.6, 7.9 Hz, 3H), 7.18 (d, *J* = 7.1 Hz, 1H), 4.81 (s, 1H), 3.96 (s, 2H), 3.49 – 3.20 (m, 2H), 2.18 (s, 2H), 2.09 (s, 2H), 1.48 (s, 9H) ppm; ^13^C NMR (75 MHz, chloroform-*d*) δ 154.3, 152.6, 149.3, 145.8, 144.0, 139.1, 136.5, 131.4, 128.7, 127.1, 127.1, 119.7, 119.3, 116.1, 110.8, 109.5, 79.2, 73.1, 30.4, 29.2, 28.0 ppm; HRMS (ESI) calcd for [C_25_H_26_BrN_5_O_3_+H]^+^ 524.1297, found 524.1295.

*tert-Butyl-4-((2-(6-(4-hydroxyphenyl)-[1,2,4]triazolo[4,3-a]pyridin-3-yl)quinolin-8-yl)oxy)piperidine-1-carboxylate* ***(9)***. To a Schlenk tube were added **8** (79.6 mg, 0.152 mmol), (4-hydroxyphenyl)boronic acid (24.45 mg, 0.182 mmol), Pd(PPh_3_)_4_ (8.77 mg, 0.0076 mmol), Na_2_CO_3_ (64.37 mg, 0.607 mmol) and a stir bar, and the tube was evacuated and backfilled with Ar three times. Then, 1 mL of 1,4-dioxane/H_2_O (4:1) was added to the tube under an Ar atmosphere. The tube was sealed and stirred at 100 °C for 6 h. The reaction mixture was filtered through Celite and concentrated under vacuum. The product was purified by chromatography (PE; 0-2% MeOH/DCM) to afford the desired product **9** as a white solid (67.5 mg, yield 62.8%). ^1^H NMR (300 MHz, DMSO-*d*_6_) δ 10.21 (s, 1H), 9.81 (s, 1H), 8.50 (s, 2H), 8.01 (s, 1H), 7.79 (s, 1H), 7.55 (s, 4H), 7.37 (s, 1H), 6.93 (s, 2H), 4.82 (s, 1H), 3.40 (s, 2H), 2.98 (s, 2H), 1.86 (s, 2H), 1.57 (s, 2H), 1.37 (s, 9H) ppm; ^13^C NMR (75 MHz, DMSO-*d*_6_) δ 158.5, 154.2, 153.0, 150.4, 146.8, 144.6, 139.6, 137.6, 130.1, 129.2, 129.0, 128.9, 128.0, 127.2, 122.7, 120.2, 116.4, 116.1, 115.8, 112.9, 79.0, 73.1, 55.3, 30.6, 28.5 ppm; HRMS (ESI) calcd for [C_31_H_31_N_5_O_4_+H]^+^ 538.2454, found 538.2452.

*4-(3-(8-(Piperidin-4-yloxy)quinolin-2-yl)-[1,2,4]triazolo[4,3-a]pyridin-6-yl)phenol* ***(MJ-1055)***. A mixture of **9** (26.9 mg, 0.05 mmol) and 4 M HCl in EtOH (2 mL) was stirred at 50 °C for 30 min. The reaction mixture was cooled to room temperature and filtered to afford the desired product MJ-1055 as a white solid (19.9 mg, yield 91.1%). ^1^H NMR (300 MHz, CD_3_OD) δ 10.68 (s, 1H), 8.59 (s, 2H), 8.44 (s, 1H), 8.24 (s, 1H), 7.67 (s, 4H), 7.48 (s, 1H), 7.07 (s, 2H), 5.05 (s, 1H), 3.11 (s, 2H), 2.82 (s, 2H), 2.30-1.90 (m, 4H) ppm; ^13^C NMR (75 MHz, CD_3_OD) δ 160.4, 153.7, 145.7, 145.4, 140.9, 139.4, 139.0, 134.6, 131.4, 130.7, 130.2, 127.7, 126.1, 121.9, 121.3, 117.6, 114.3, 112.4, 70.4, 49.8, 49.5, 49.3, 49.0, 48.7, 48.4, 48.1, 41.5, 28.1 ppm; HRMS (ESI) calcd for [C_26_H_24_N_5_O_2_+H]^+^ 438.1926, found 438.1930. HPLC analysis: MeOH containing 1‰ CF_3_COOH /H_2_O (80:20), 6.42 min, 97.9% purity.

**9. ^1^H NMR spectra**

*2-Chloro-5-(3-methoxyphenyl)-5H-pyrrolo[3,2-d]pyrimidine* ***(1)***

***
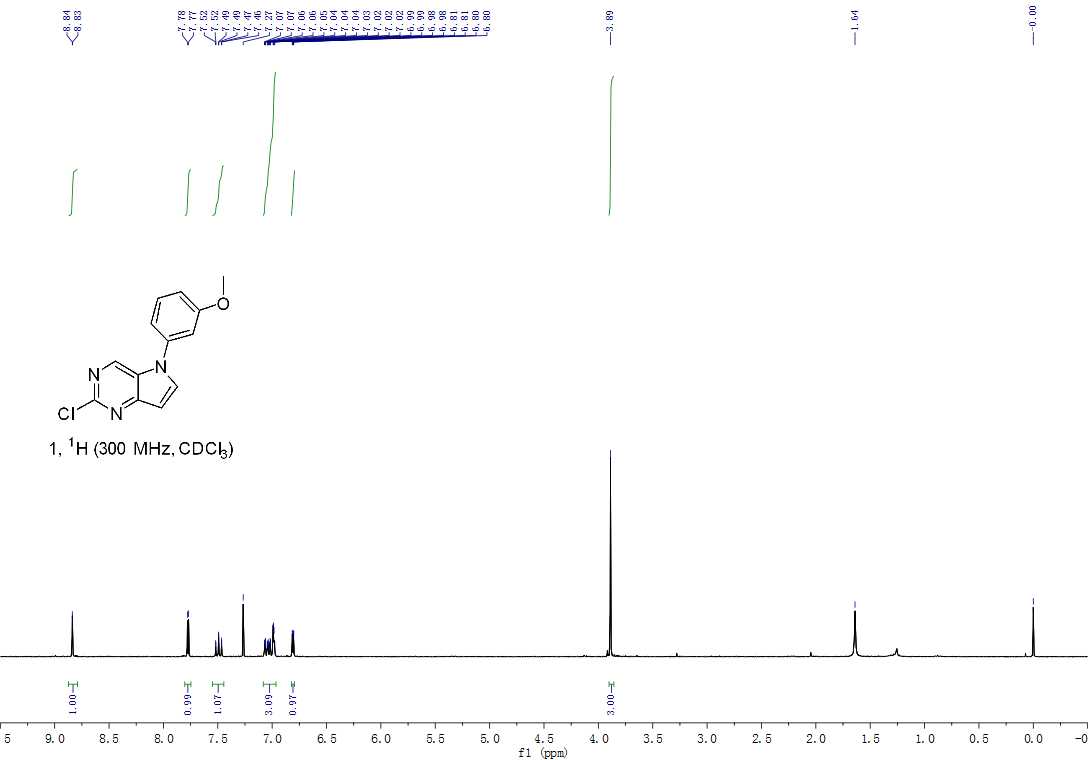
***

*tert-Butyl-4-(4-((5-(3-methoxyphenyl)-5H-pyrrolo[3,2-d]pyrimidin-2-yl)amino)phenyl)piperazine-1-carboxylate* ***(2)***

***
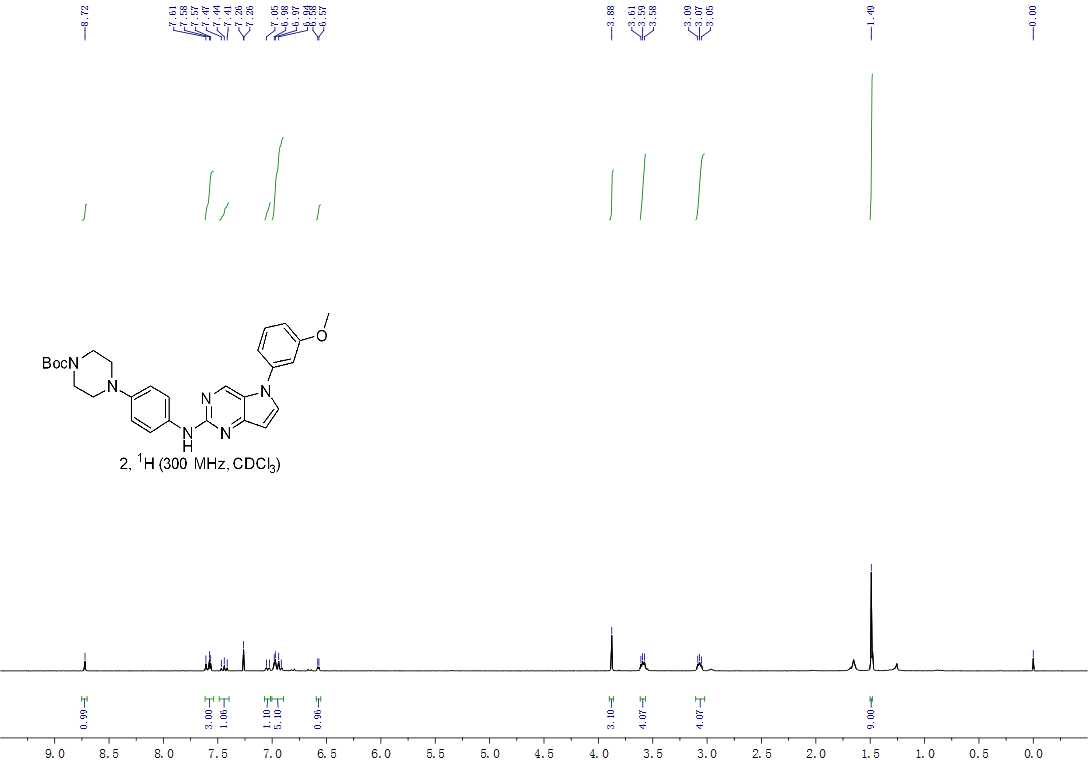
***

*5-(3-Methoxyphenyl)-N-(4-(piperazin-1-yl)phenyl)-5H-pyrrolo[3,2-d]pyrimidin-2-amine* ***(MJ-4)*** *hydrochloride*.


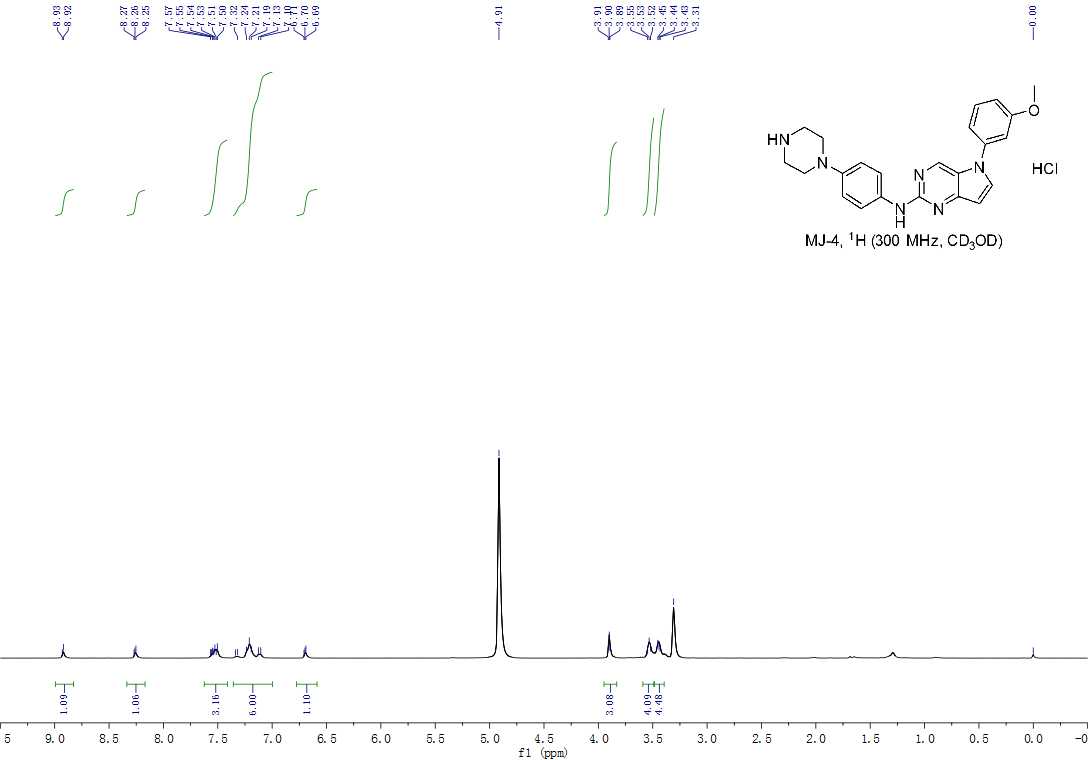


*2-Chloro-5-iodo-7-isopropyl-7H-pyrrolo[2,3-d]pyrimidine* ***(3)***

***
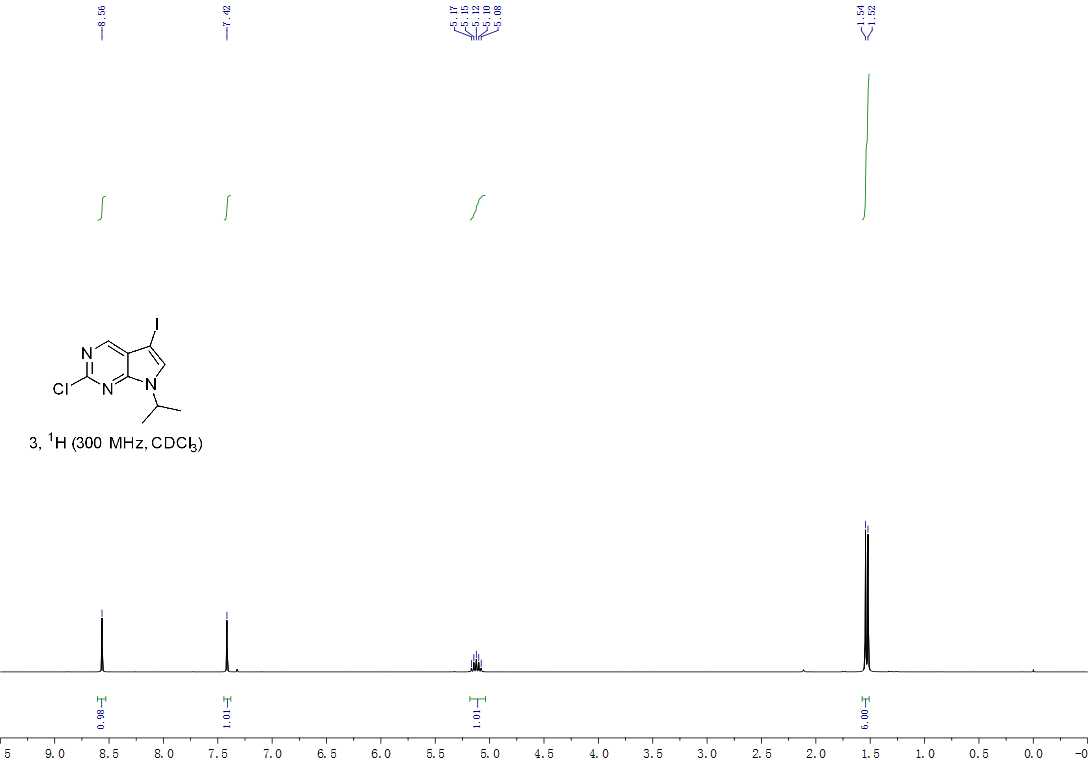
***

*2-Chloro-7-isopropyl-7H-pyrrolo[2,3-d]pyrimidine-5-carbonitrile* ***(4)***

***
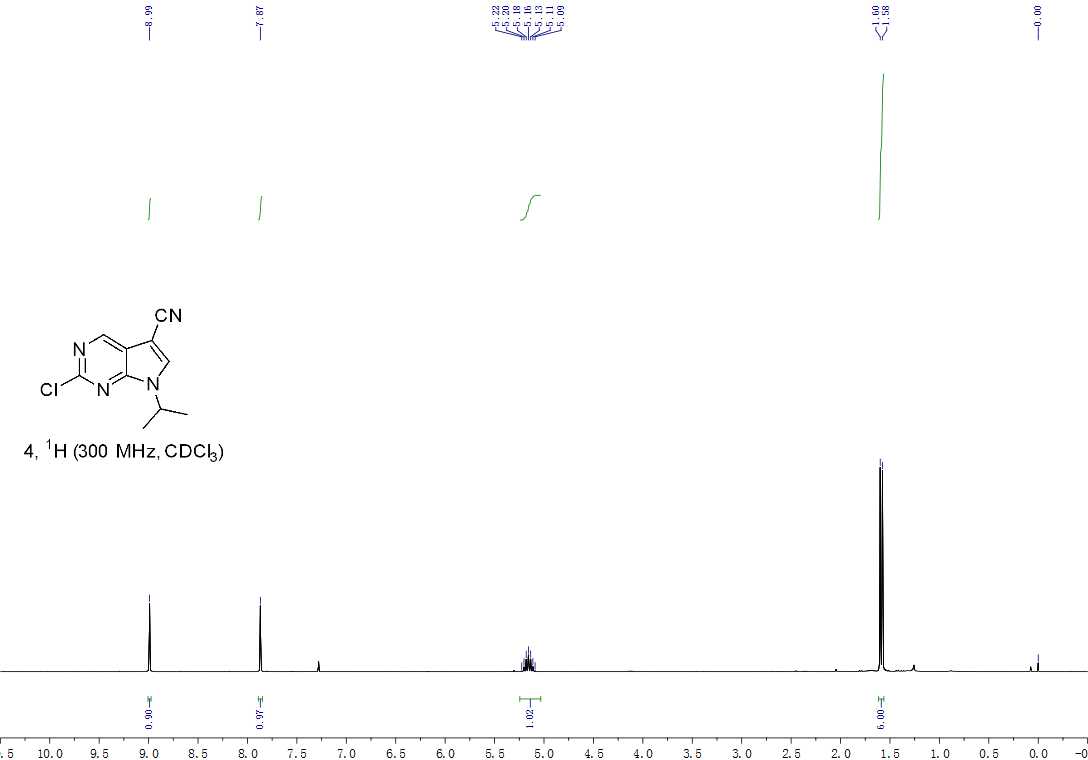
***

*tert-Butyl-4-(4-((5-cyano-7-isopropyl-7H-pyrrolo[2,3-d]pyrimidin-2-yl)amino)phenyl)piperazine-1-carboxylate* ***(5)***

***
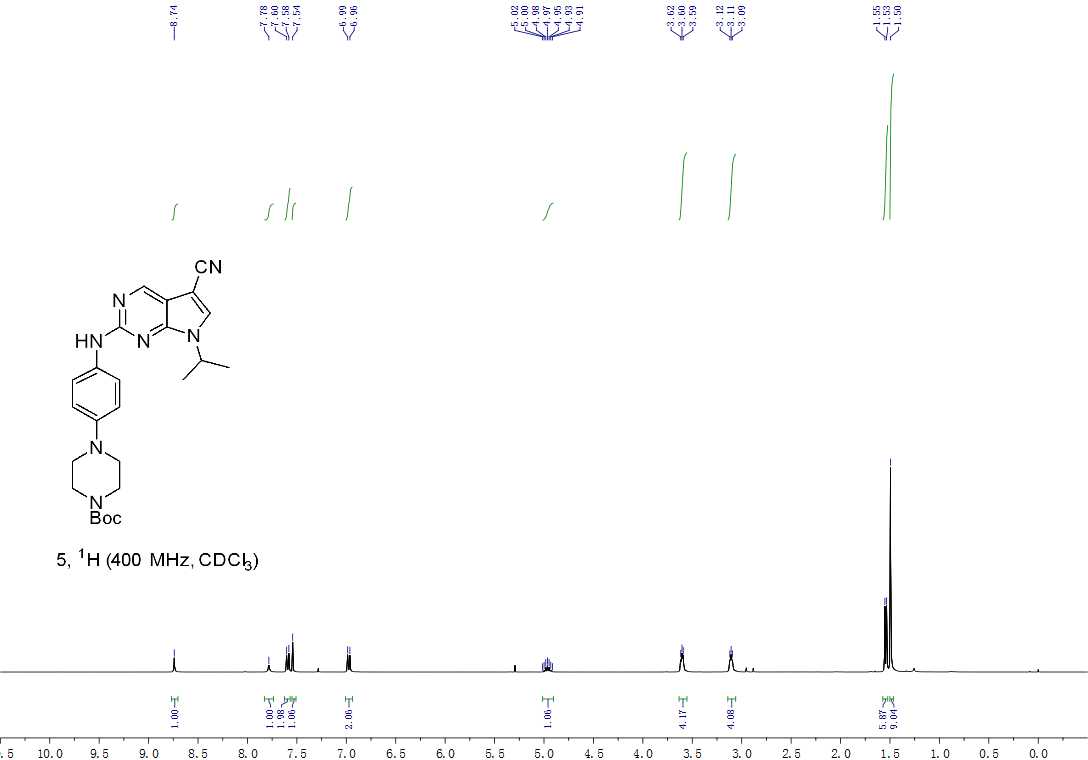
***

*tert-Butyl-4-(4-((5-carbamoyl-7-isopropyl-7H-pyrrolo[2,3-d]pyrimidin-2-yl)amino)phenyl)piperazine-1-carboxylate* ***(6)***

***
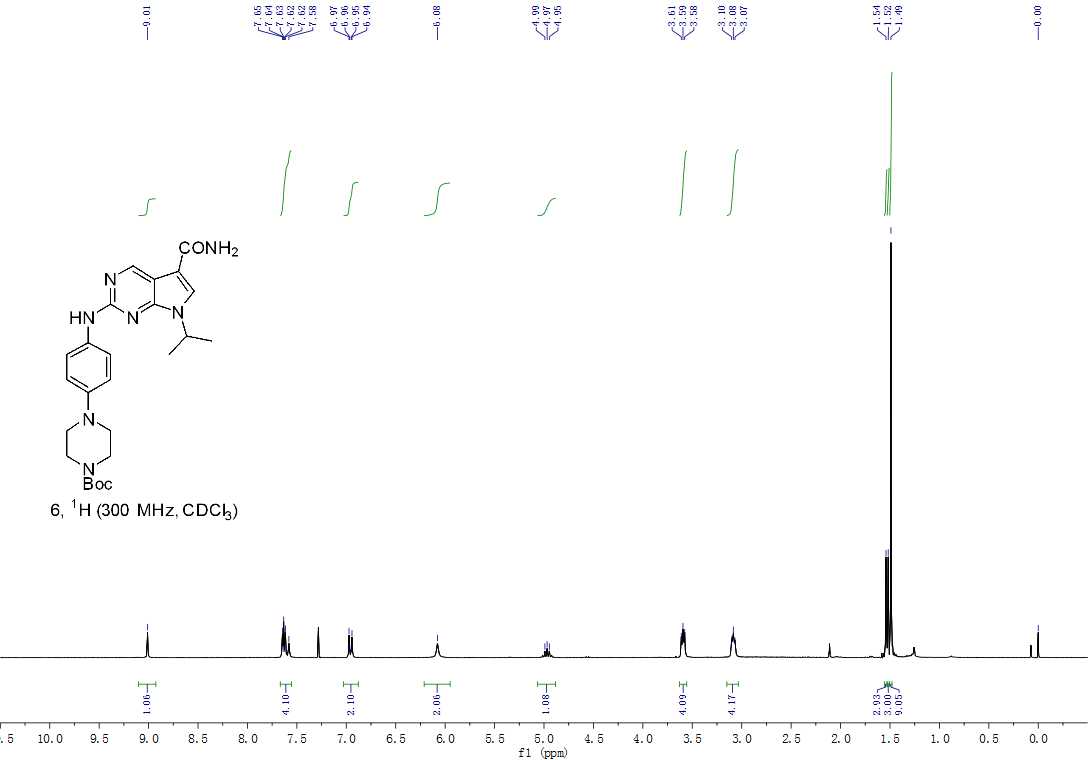
***

*7-Isopropyl-2-((4-(piperazin-1-yl)phenyl)amino)-7H-pyrrolo[2,3-d]pyrimidine-5-carboxamide* ***(MJ-115)*** *hydrochloride*

*
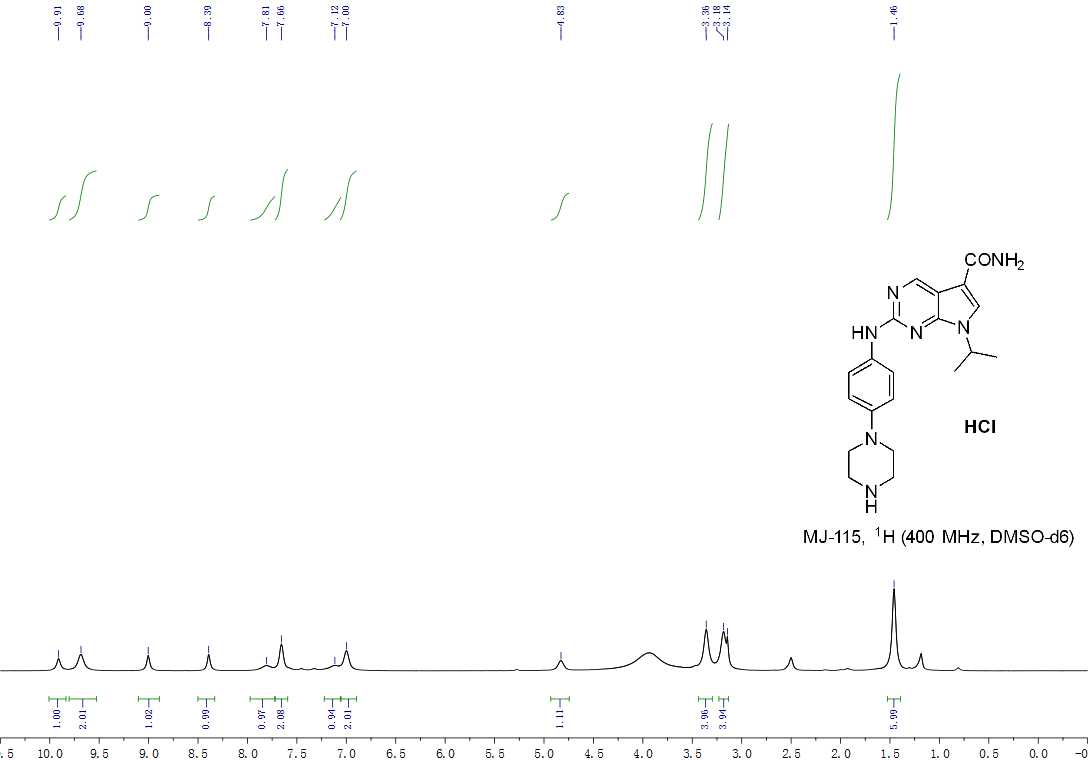
*

*2-(6-Bromo-[1,2,4]triazolo[4,3-a]pyridin-3-yl)quinolin-8-ol* ***(7)***

***
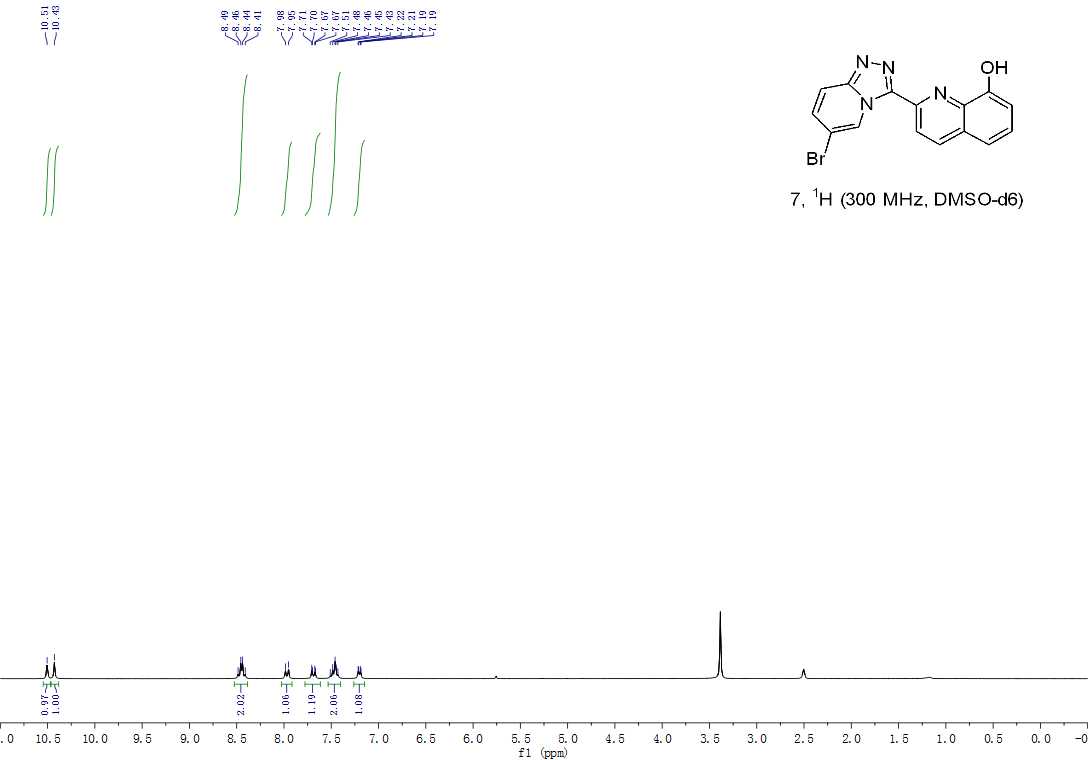
***

*tert-Butyl-4-((2-(6-bromo-[1,2,4]triazolo[4,3-a]pyridin-3-yl)quinolin-8-yl)oxy)piperidine-1-carboxylate* ***(8)***

***
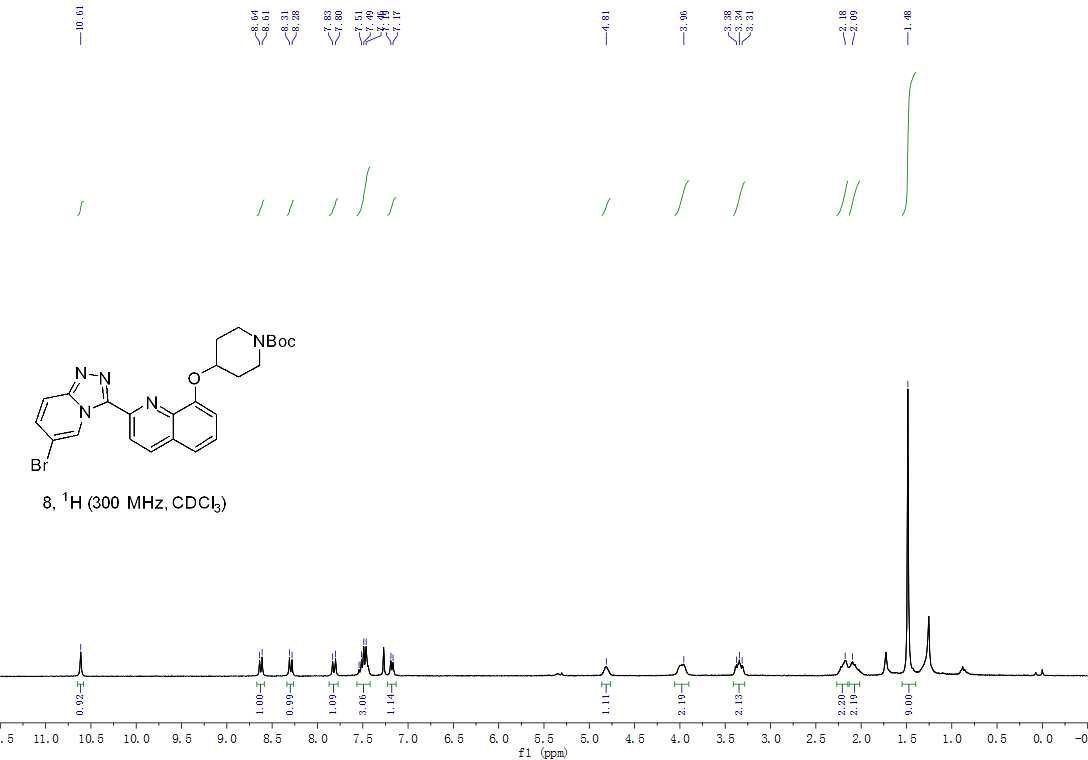
***

*tert-Butyl-4-((2-(6-(4-hydroxyphenyl)-[1,2,4]triazolo[4,3-a]pyridin-3-yl)quinolin-8-yl)oxy)piperidine-1-carboxylate* ***(9)***

***
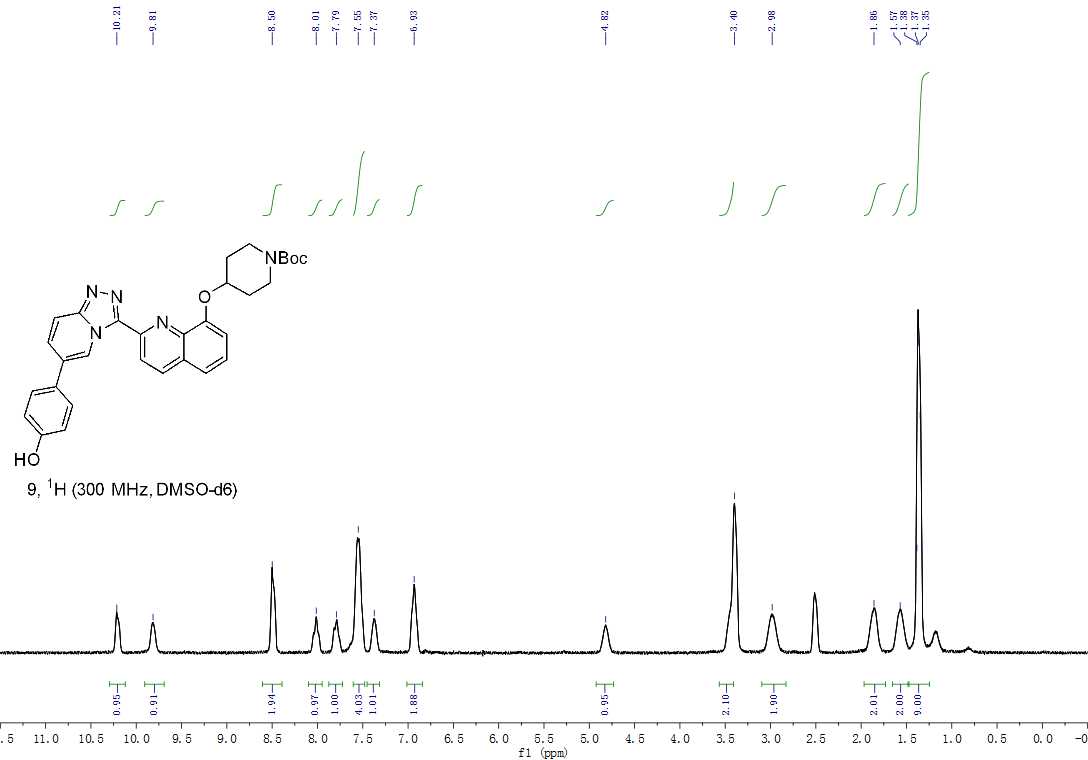
***

*4-(3-(8-(Piperidin-4-yloxy)quinolin-2-yl)-[1,2,4]triazolo[4,3-a]pyridin-6-yl)phenol* ***(MJ-1055)***

***
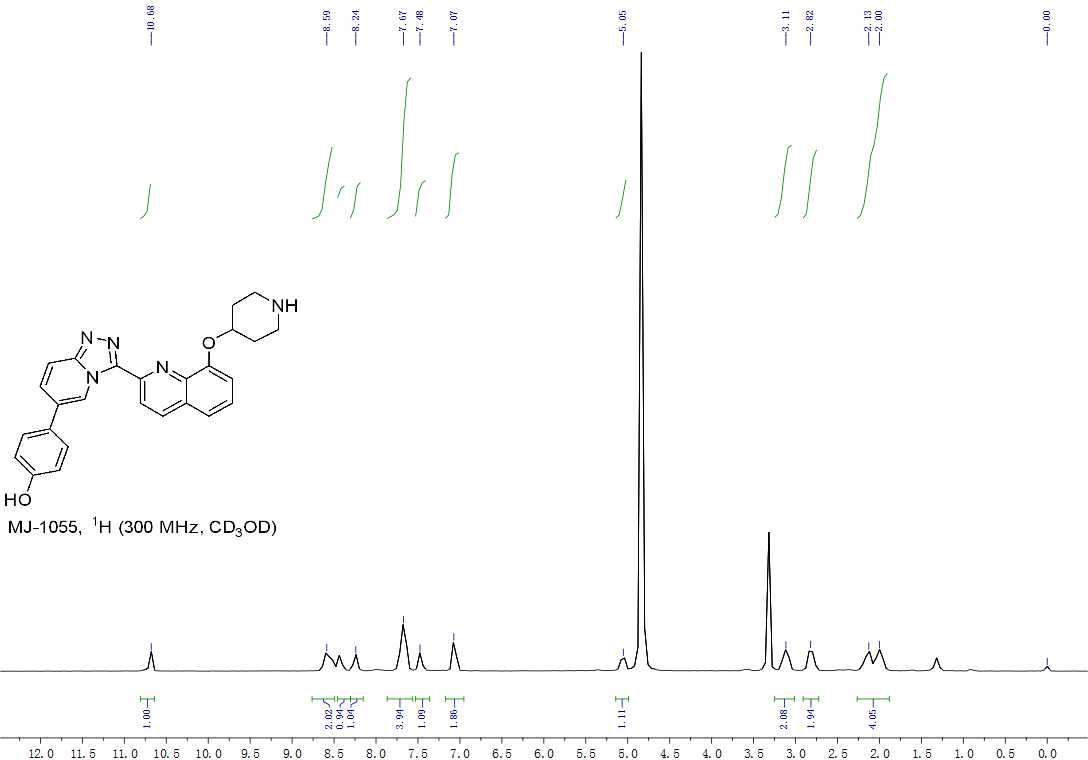
***

**10. ^13^C NMR spectra**

*2-Chloro-5-(3-methoxyphenyl)-5H-pyrrolo[3,2-d]pyrimidine* ***(1)***

***
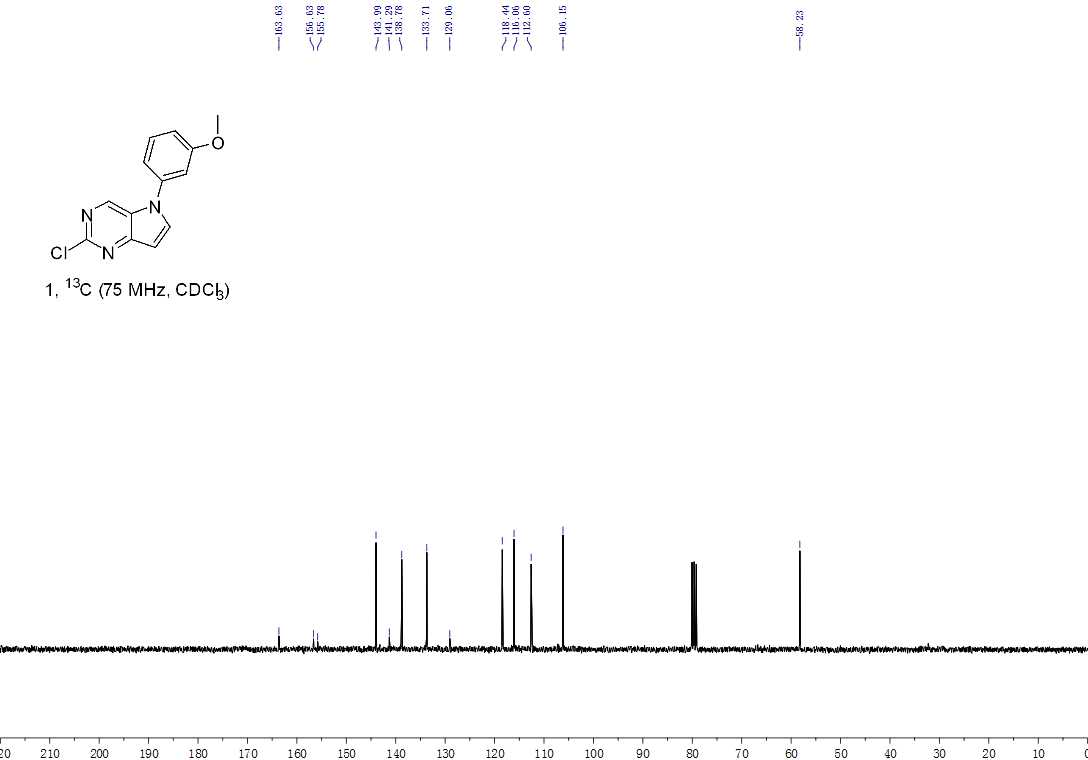
***

*tert-Butyl-4-(4-((5-(3-methoxyphenyl)-5H-pyrrolo[3,2-d]pyrimidin-2-yl)amino)phenyl)piperazine-1-carboxylate* ***(2)***

***
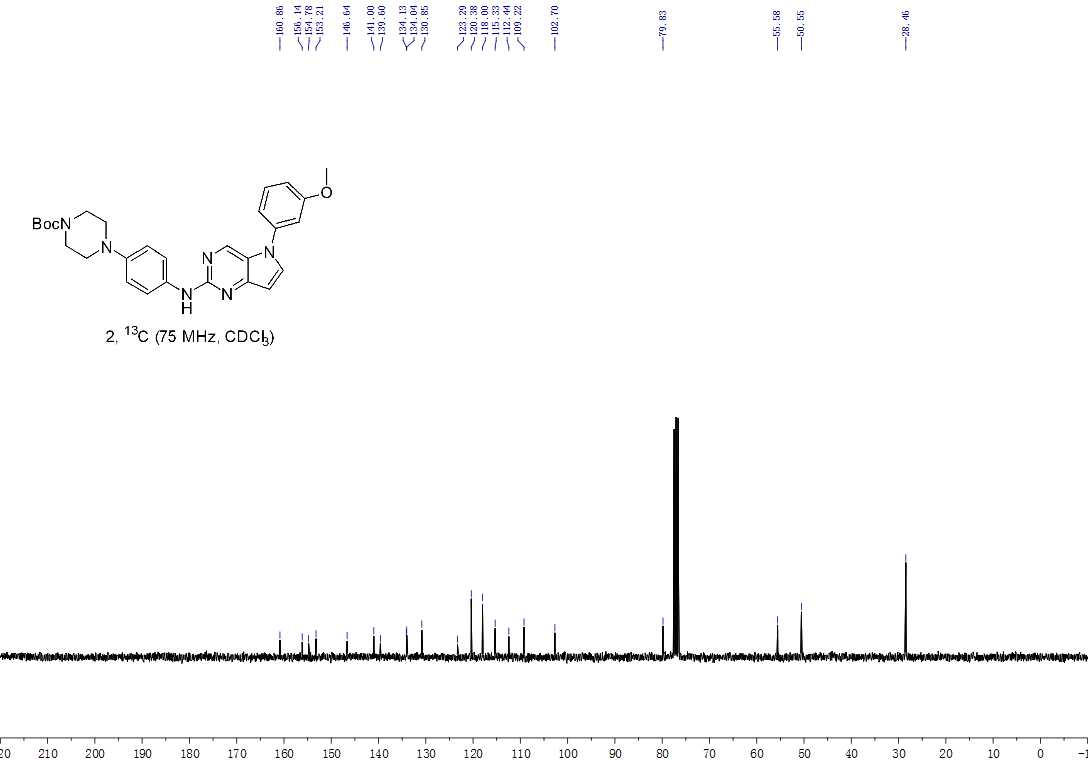
***

*5-(3-Methoxyphenyl)-N-(4-(piperazin-1-yl)phenyl)-5H-pyrrolo[3,2-d]pyrimidin-2-amine* ***(MJ-4)*** *hydrochloride*.


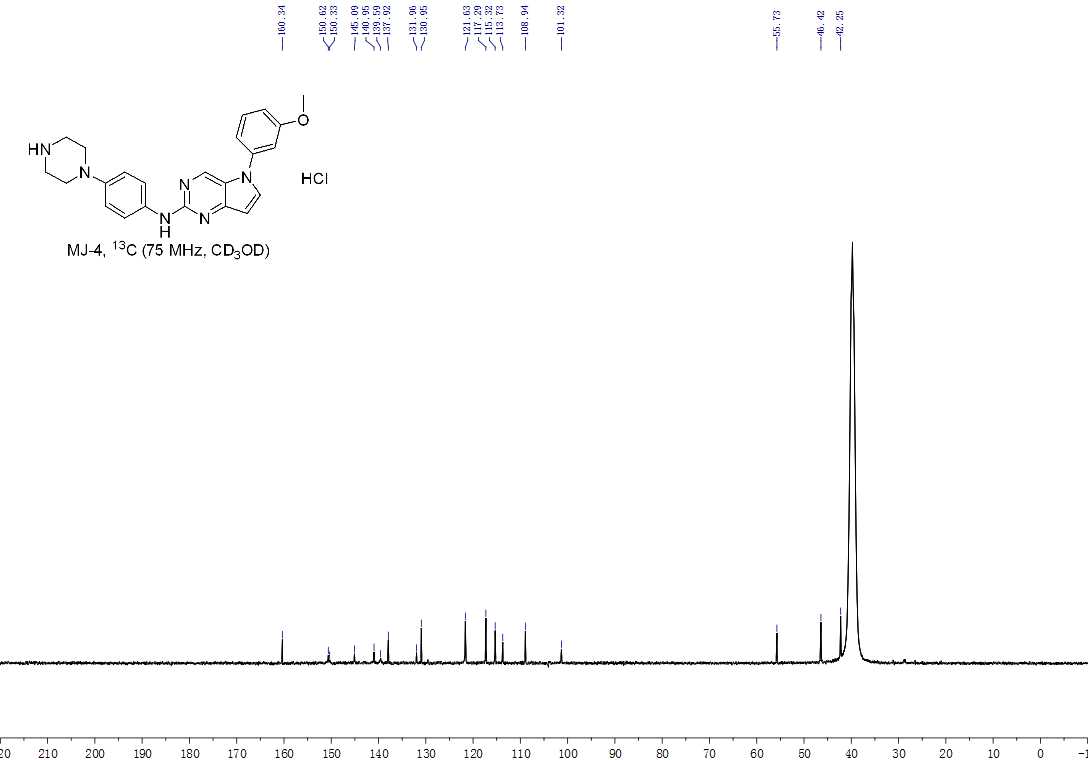


*2-Chloro-5-iodo-7-isopropyl-7H-pyrrolo[2,3-d]pyrimidine* ***(3)***

***
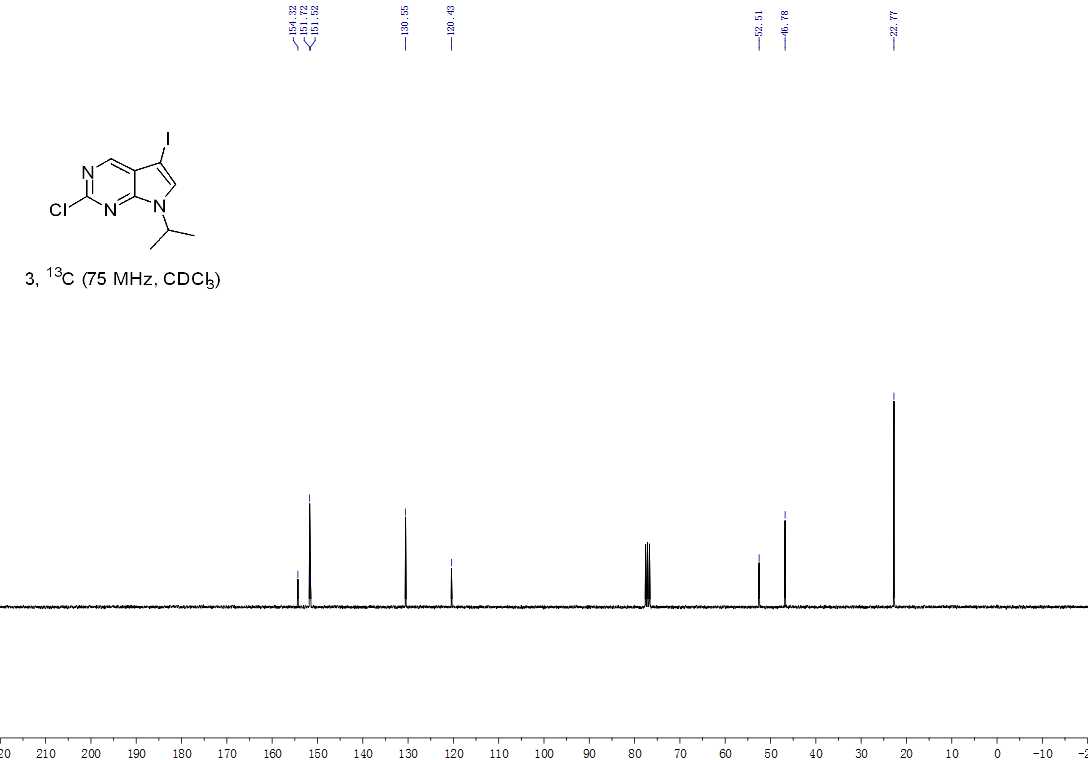
***

*2-Chloro-7-isopropyl-7H-pyrrolo[2,3-d]pyrimidine-5-carbonitrile* ***(4)***

***
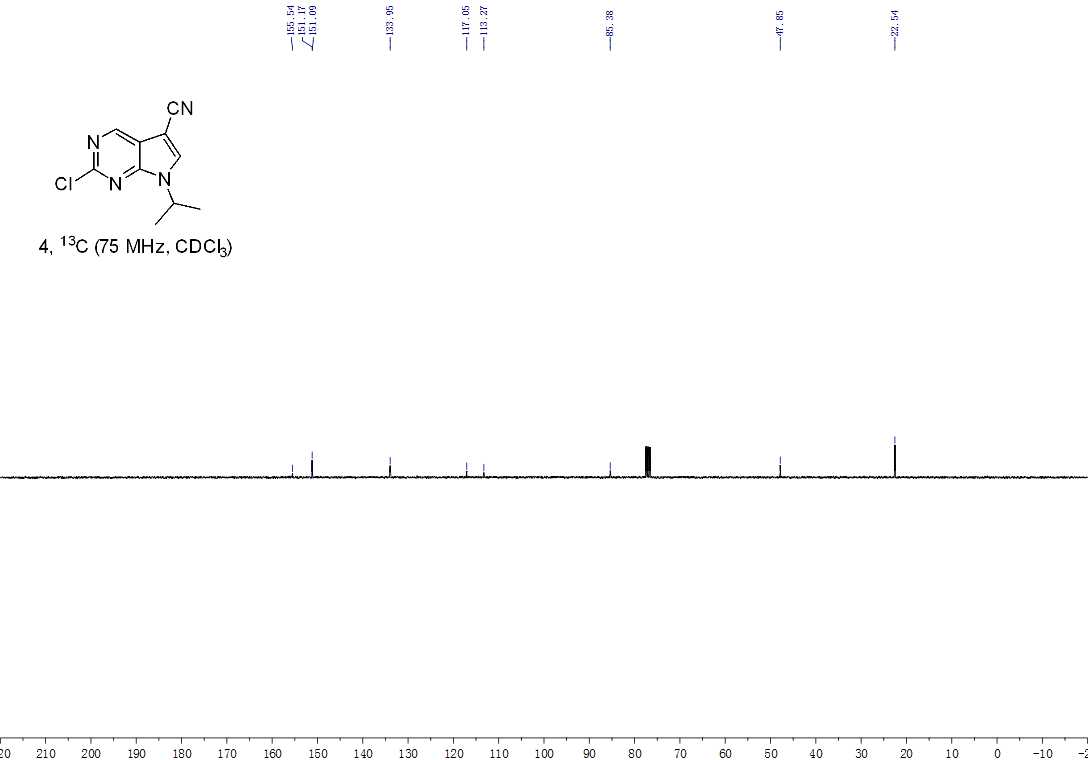
***

*tert-Butyl-4-(4-((5-cyano-7-isopropyl-7H-pyrrolo[2,3-d]pyrimidin-2-yl)amino)phenyl)piperazine-1-carboxylate* ***(5)***

***
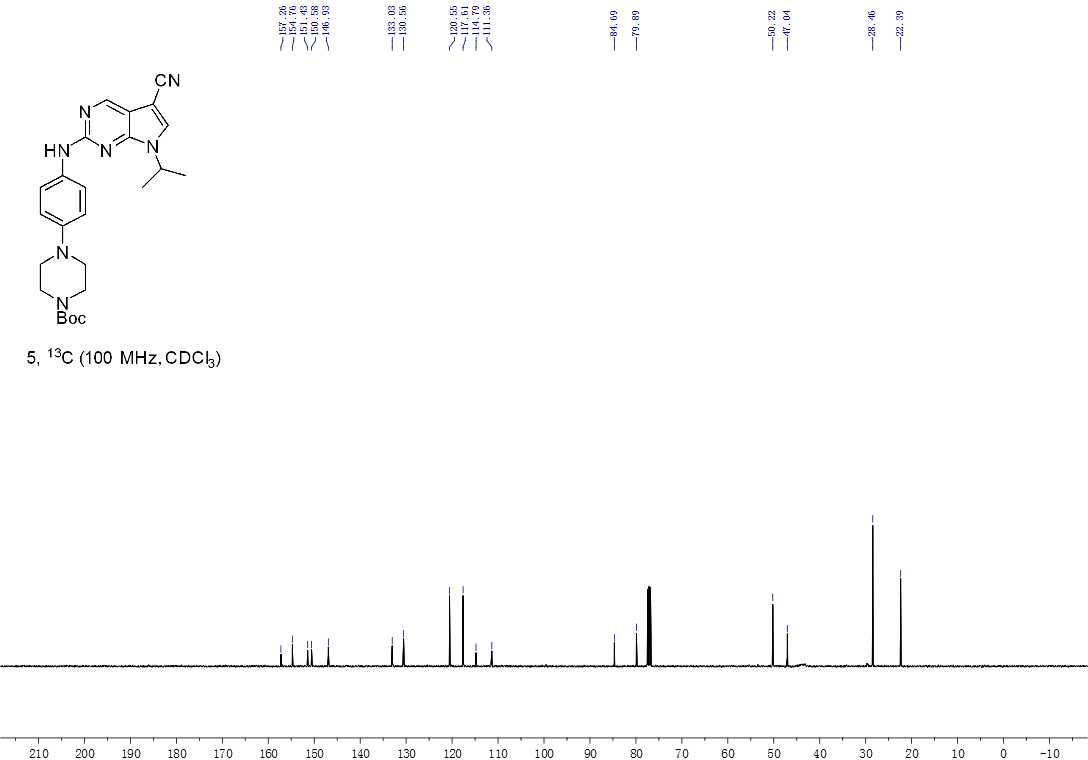
***

*tert-Butyl-4-(4-((5-carbamoyl-7-isopropyl-7H-pyrrolo[2,3-d]pyrimidin-2-yl)amino)phenyl)piperazine-1-carboxylate* ***(6)***

***
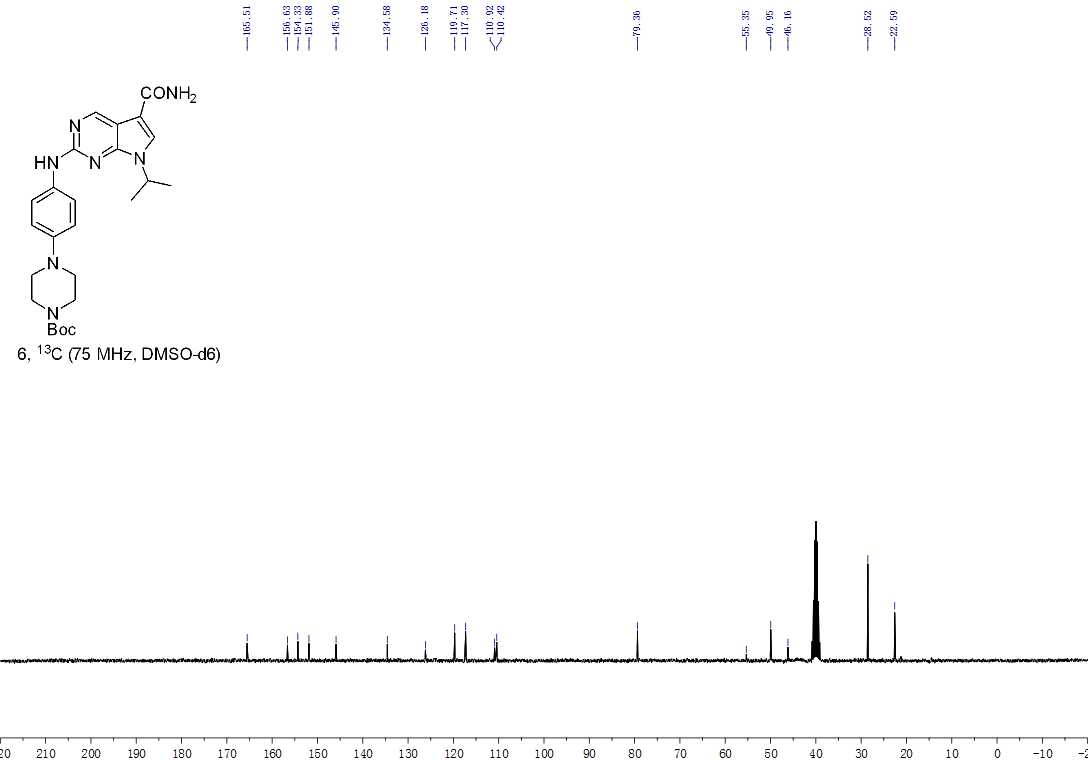
***

*7-Isopropyl-2-((4-(piperazin-1-yl)phenyl)amino)-7H-pyrrolo[2,3-d]pyrimidine-5-carboxamide* ***(MJ-115)*** *hydrochloride*

*
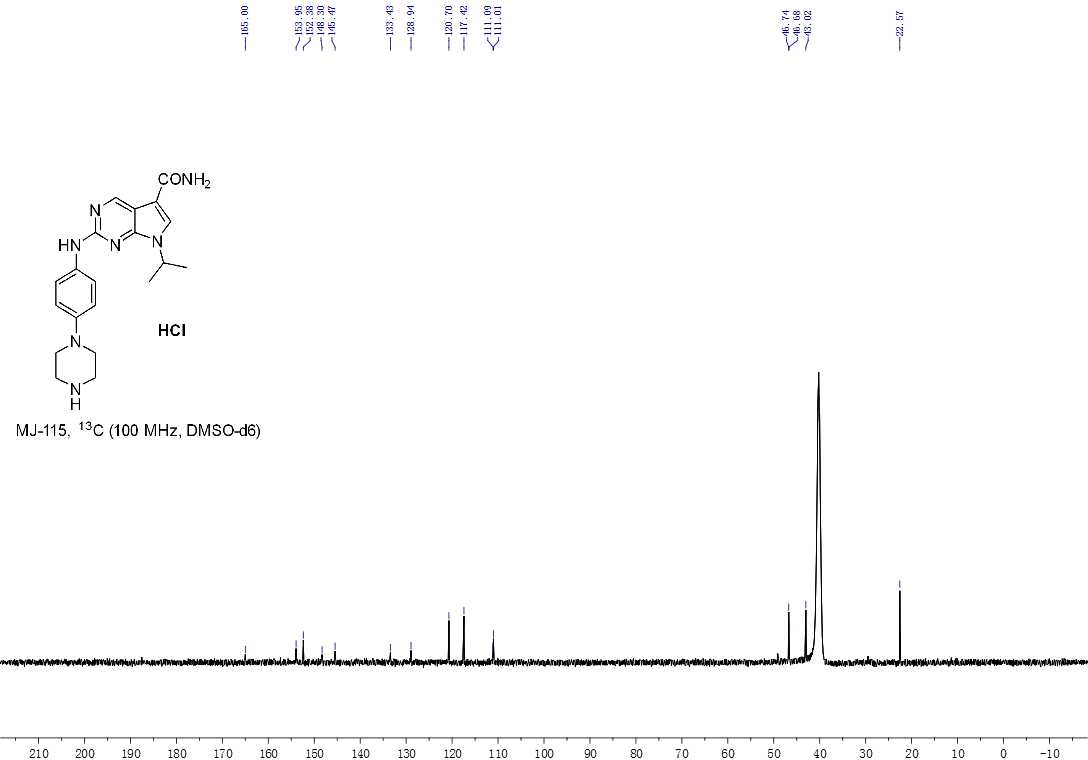
*

*2-(6-Bromo-[1,2,4]triazolo[4,3-a]pyridin-3-yl)quinolin-8-ol* ***(7)***

***
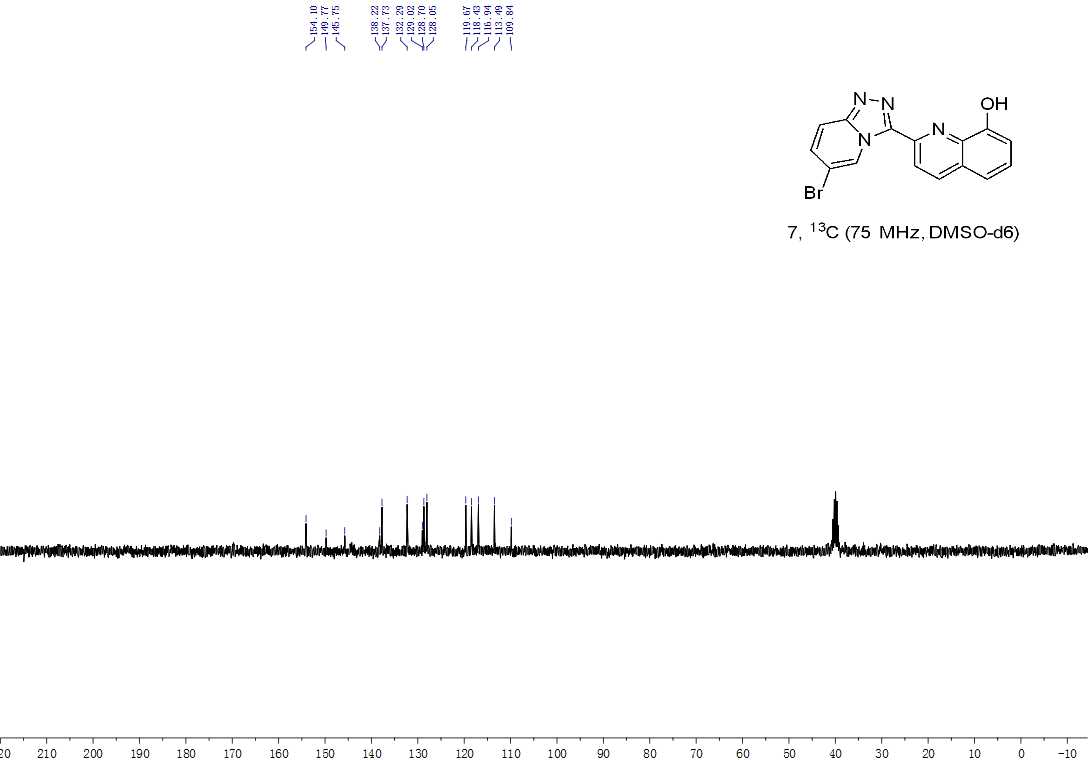
***

*tert-Butyl-4-((2-(6-bromo-[1,2,4]triazolo[4,3-a]pyridin-3-yl)quinolin-8-yl)oxy)piperidine-1-carboxylate* ***(8)***

***
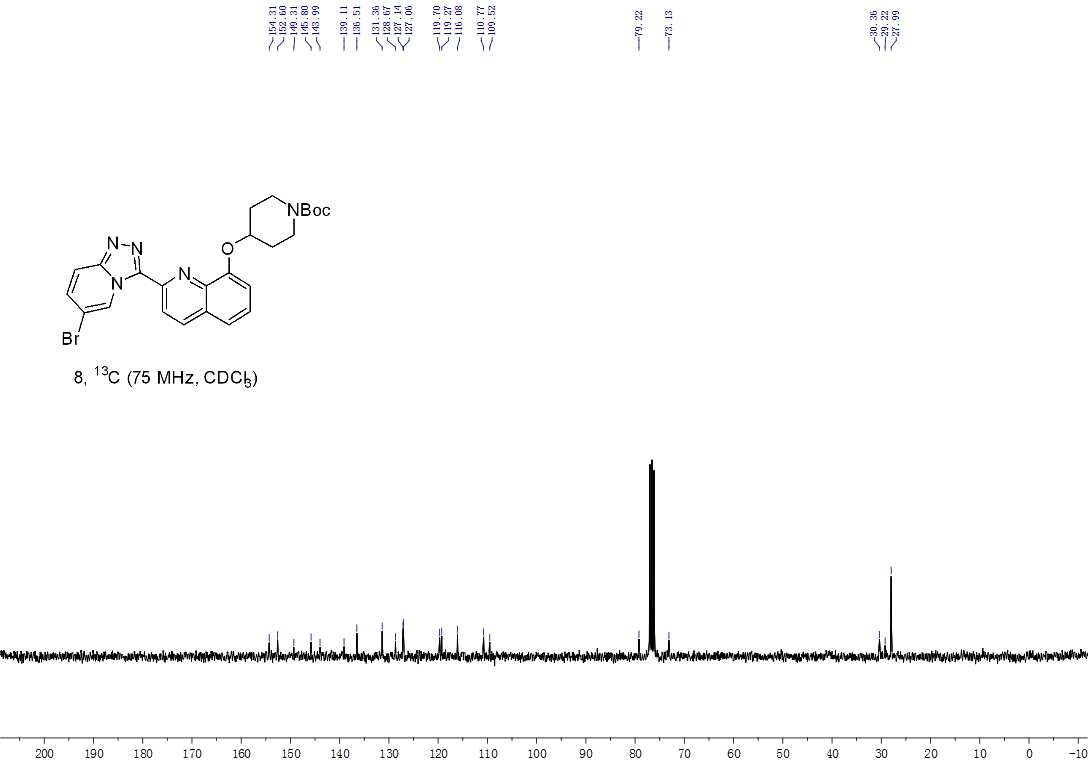
***

*tert-Butyl-4-((2-(6-(4-hydroxyphenyl)-[1,2,4]triazolo[4,3-a]pyridin-3-yl)quinolin-8-yl)oxy)piperidine-1-carboxylate* ***(9)***

***
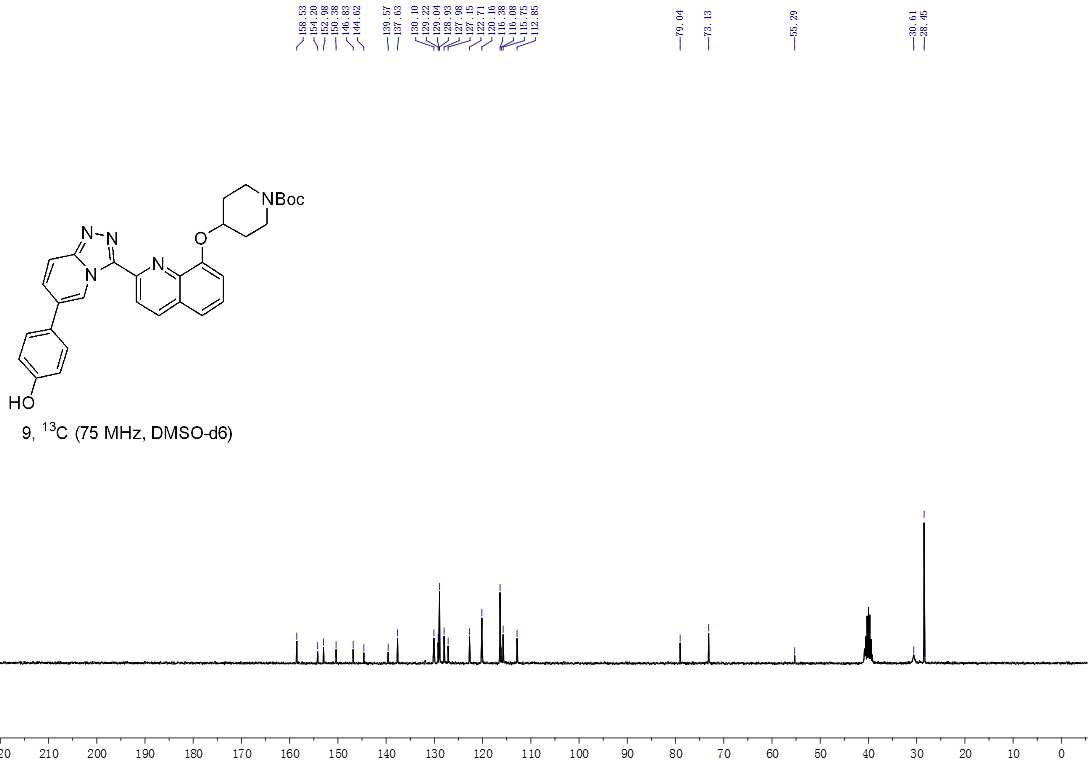
***

*4-(3-(8-(Piperidin-4-yloxy)quinolin-2-yl)-[1,2,4]triazolo[4,3-a]pyridin-6-yl)phenol* ***(MJ-1055)***

**
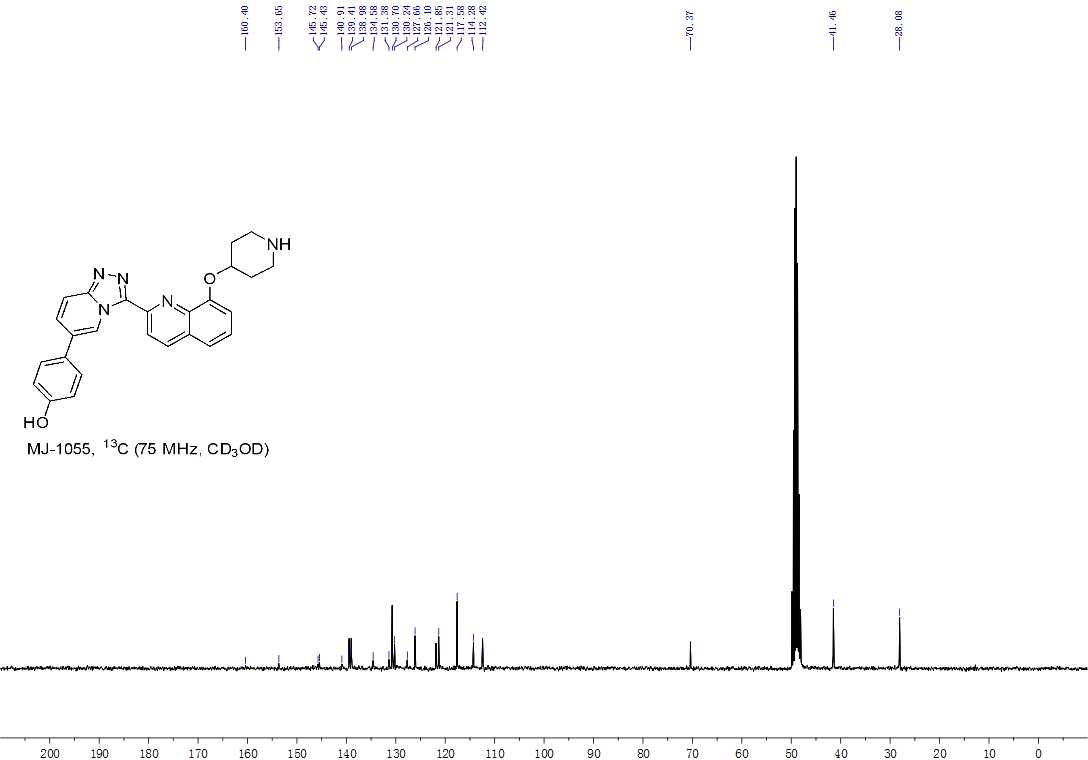
**

**References**

1. Allen S, Celeste LL, Davis GT, Delisle RK, Hicks JM, Gross SD, Hicken EJ, Jackson LJ, Kallan NC, Lyssikatos JP, Marmsater FP, Munson MC, Pheneger J, Rast B, Robinson JE, Schlachter ST, Topalov GT, Wright DA, Zhao Q (2015) Triazolopyridine compounds as PIM kinase inhibitors. US Patent 8,987,251, 24 Mar 2015.
